# Real-Time Human Ambulation, Activity, and Physiological Monitoring: Taxonomy of Issues, Techniques, Applications, Challenges and Limitations

**DOI:** 10.3390/s131012852

**Published:** 2013-09-25

**Authors:** Rinat Khusainov, Djamel Azzi, Ifeyinwa E. Achumba, Sebastian D. Bersch

**Affiliations:** School of Engineering, Faculty of Technology, University of Portsmouth, Anglesea Building, Anglesea Road, Portsmouth PO1 3DJ, UK; E-Mails: Rinat.Khusainov@port.ac.uk (R.K.); Djamel.Azzi@port.ac.uk (D.A.); Sebastian.Bersch@port.ac.uk (S.D.B.)

**Keywords:** sensor-based monitoring, sensor placement, monitoring device framework, data collection and processing, gait assessment/fall risk estimation

## Abstract

Automated methods of real-time, unobtrusive, human ambulation, activity, and wellness monitoring and data analysis using various algorithmic techniques have been subjects of intense research. The general aim is to devise effective means of addressing the demands of assisted living, rehabilitation, and clinical observation and assessment through sensor-based monitoring. The research studies have resulted in a large amount of literature. This paper presents a holistic articulation of the research studies and offers comprehensive insights along four main axes: distribution of existing studies; monitoring device framework and sensor types; data collection, processing and analysis; and applications, limitations and challenges. The aim is to present a systematic and most complete study of literature in the area in order to identify research gaps and prioritize future research directions.

## 1. Introduction

The proportion of the population requiring healthcare services and assisted living is increasing due to population aging resulting from higher life expectancy [1]. This has catalyzed increase in research studies focused on automated monitoring of mobility, Activities of Daily Living (ADL), and physiological (vital) signs of elderly adults living independently in their homes. The traditional monitoring method is physical observation, which is costly and increasingly infeasible in view of population aging. It places a huge burden on service providers. Consequently, sensor-based real-time monitoring has been a subject of recent research studies. The general aim is to devise effective and automated means of addressing the demands of assisted living, rehabilitation, and wellness evaluation through sensor-based monitoring. The acquired sensor data are often processed and analyzed for various applications including *gait assessment*, *fall risk estimation*, *fall detection*, *ADL recognition/classification* and *energy expenditure estimation*.

The methods and mechanics of human ambulation are collectively referred to as *gait* which consists of a cycle of ‘controlled falls’ [2]. Gait assessment is a method and assistive clinical tool used to systematically measure and characterize human locomotion. It is applied in rehabilitation and diagnosis of medical conditions [3]. Gait dysfunction is listed as a fall risk factor such that *fall risk estimation* often involves measurements of gait parameters such as imbalance, postural sway, and functional reach, *etc.* Fall risk factors are mostly either of environmental or physiological sources. One-third of falls by elderly adults involve environmental hazards in the home, the most common being tripping or stumbling over objects [4]. Environmental fall risk factors are generally detectable by physical observation, while physiological factors are traditionally assessed with clinical instruments and scores. A current trend is assessment of physiological risk factors from sensor data. The activation of a fall risk factor(s) precipitates a *fall*. Human fall is defined in this context as unintentional and uncontrolled downwards motion.

*Activities of Daily Living* (ADL) encompass a set of activities and tasks, which subjects undertake routinely in their everyday life. The initial set of ADL (IndexADL) was developed in [5] for a study of results of treatment and prognosis in elderly and chronically ill. Another set, the Physical Self-Maintenance Scale (PSMS), was proposed in [6] for functional assessment of elderly adults. The PSMS, which is only marginally different from IndexADL, was complemented by the Instrumental Activities of Daily Living (InstrumentalADL) proposed in [7]. These have evolved into standard sets of activities used by medical professionals and healthcare service providers to assess well-being and the need for assisted living and rehabilitation [8].

ADL performance generally entails *Energy Expenditure* (EE). The daily EE of a subject consists of three different separately estimable components: Basal Metabolic Rate (BMR, the minimum amount of energy a body requires when lying in physiological and mental rest), Diet Induced Thermogenesis (DIT), and Physical Activity (PA). EE is used to estimate the effect of healthcare interventions and for the prevention and management of certain diseases (e.g., degenerative diseases) based on the notion that physical activity may prevent or delay the onset or progress of the disease. EE is also used to assess patients' healthy daily lifestyle such as metabolic requirements, fuel utilization, thermic effect of foods, drinks, drugs, and emotional state [9]. Energy expenditure is also theorized to be physiologically related to mechanisms of diabetes.

Research studies on sensor-based monitoring of subjects for these various applications have resulted in a large amount of literature including survey study reports [10–15]. The existing survey study reports are mostly based on a review of relatively small numbers of literature items. In addition, those survey studies are limited in scope and details, and with focus areas that are different from those presented in this paper. This paper presents a holistic articulation of literature on sensor-based monitoring of mobility and activities of daily living and offers comprehensive insights along four main axes: applications, monitoring device framework and sensor types; distribution of existing studies with respect to applications and sensor types; data collection, processing and analysis; and research gaps, limitations and challenges. The aim is to present a systematic and the most complete study of literature in the area in order to facilitate the identification of research gaps and prioritize future research directions.

The rest of the paper is organized as follows: Section 2 details monitoring device platform and sensor types; Section 3 presents the distribution of existing studies with respect to different applications and sensor types; Section 4 addresses sensor placement and data collection; Section 5 addresses data processing for feature extraction and selection; Section 6 details the various application areas; Section 7 highlights research gaps and possible future research directions; Section 8 articulates the challenges and ethical issues; and Section 9 concludes the paper.

## 2. Monitoring Device Platform and Sensor Types

**Figure 1.**
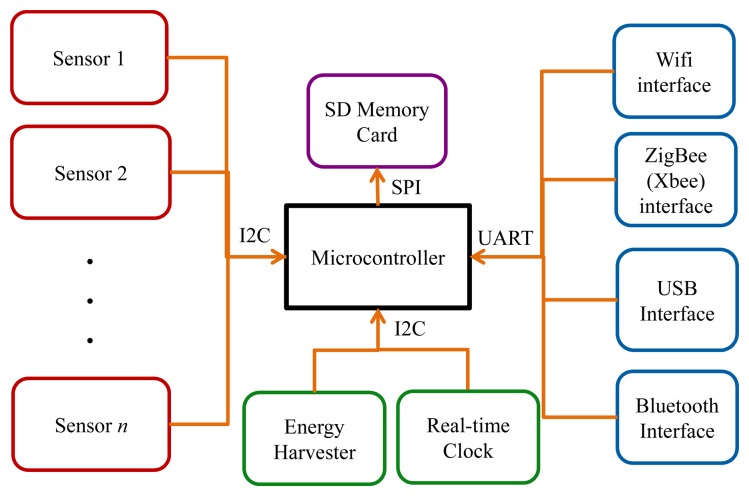
Monitoring device platform.

Figure 1 presents a monitoring device framework combining features from existing platforms and consisting of a microcontroller, sensors, memory card, energy harvester and communication interfaces. The microcontroller is the processing unit and integrates power and communication management functions. It also hosts the necessary software. The communication interface could be based on any of the four basic communication standards (Zigbee or Xbee, Bluetooth, USB, and WiFi). Memory and Energy Harvester are optional. Energy Harvester is used to extend the finite uptime of the monitoring devices, which commonly use batteries with limited operational time. It transforms user movements and body heat into energy to charge the batteries.

Often, the design aim of a monitoring device is to meet the requirements of small size, unobtrusiveness, light weight, wearability, multi-sensor platform, low power consumption, and wired/ wireless communication. The USB (wired) interface is generally used for connecting the device to a computer in order to program the microcontroller. The platform facilitates the synergy of different sensors in order to extend the spatial and temporal sensing coverage for sensor fusion [16]. The type of sensor(s) used depends on the selected monitoring technique, which can be visual and non-visual. Visual monitoring often uses cameras installed in fixed positions in the monitored environment with or without markers placed on the subject. This poses a number of challenges. According to [17], twists and rotations of the human skeleton could introduce inconsistencies in motion estimation. Furthermore, cluttered scenes and/or variable lighting could distract visual attention. Rotated joints or overlapped body parts may not be detected, and markers can shift positions or come off completely [17]. The advantages of this technique include conciseness and objectivity. Its main disadvantages are high level of intrusiveness (privacy violations) and line-of-sight problem. The authors in [18] stated that “there is strong resistance from older people against the deployment of cameras in homes due to privacy concerns”. The authors in [19] proposed a privacy preserving visual monitoring approach using RGBD cameras with data analysis based on 3D depth information. The system is such that if the subject is out of the range of the 3D camera, the RGB camera is employed to continue monitoring. The release of software development kit (SDK) for Kinect cameras [20] by Microsoft has in recent years catalyzed research studies on ambulation and activity events recognition based on depth images [21–23]. The SDK facilitates skeletal tracking, “the ability to track the skeleton image of one or two people moving within the Kinect field of view” [20]. Visual monitoring will not be addressed further in this context as older people are deeply concerned about privacy issues with cameras. Also, there are space limitations and ethical issues (as highlighted in Section 7.3).

Non-visual monitoring employs a wide range of sensor types including kinematic/inertial (e.g., accelerometer, gyroscope, inclinometer, pedometer), mechanical force (e.g., ball/tilt/foot switch, sole pressure switch, force sensitive resistor), acoustic/audio (e.g., a microphone), optical (e.g., infrared sensors), radio frequency (e.g., RFID, Ubisense Real-Time Location Systems (RTLS)), atmospheric (e.g., barometer, humidity), electrical (e.g., Electromyogram (EMG), Electrocardiogram (ECG), Electrooculography (EOG)), magnetic (e.g., magnetometer), photoelectric (e.g., oximeter), and chemical/electrochemical (e.g., actinometer) sensors. These sensors have been used individually or in combination for various applications. Accelerometers provide *x* (vertical axis), *y* (sagittal axis), and *z* (frontal axis) linear motion sensing. Gyroscopes sense the rate of change of orientation (angular velocity). Inclinometers and tilt sensors give a measure of tilt angle, elevation, or depression with respect to gravity. Acoustic sensors measure vibration and sound signals, and may be ineffective in noisy environments. The Radio Frequency sensors, RFID and Ubisense RTLS, are tag-based. RFID tags are often located on target items within the monitoring environment, while the reader is usually worn on a hand (in a glove) by the subject [24,25]. The reader detects a tag's unique identifier when in contact or within close proximity. Ubisense tags can also be worn by the subject, while the Receivers (located within the monitoring environment) receive UltraWideBand (UWB) pulses from the tags in order to determine the location of the subject using 3D trilateration. The electrical sensors, EMG and ECG, measure the intensity of muscle activity in the lower extremities and the electrical activity of the heart over a period of time, respectively. EOG sensors (electrodes usually placed on the skin on either side of the eyes) measure eye position and movements. Analyses of EOG signals yield eye movement features, such as saccades, fixations, and blinks, as well as deliberate movement patterns [26]. Passive Infrared (PIR) sensors measure changes in infrared energy levels radiated by a human body often with a wavelength of between 9 and 10 μm [27]. A Pressure Mat (PM) acts as a switch generating a binary signal when pressure is applied. Its sensitivity ranges from 0.14 to 2.2 kg/cm^2^ of pressure with an active area of up to 0.21 m^2^ [27]. The use of both PIR and PM is based on mobility, where PIR can monitor the occupancy of a particular location (such as a room), while PM indicates the use of furniture and movement across doorway thresholds. The distribution of existing studies with respect to the different applications and sensor types is presented in the next section.

Sensors characteristics could contribute to study limitations and challenges if not mitigated. For instance, accelerometers are subject to fluctuation of offsets and measurement noise, while gyroscopes suffer from zero bias drift. Magnetic objects interfere with magnetometer signals, while vector magnetometers are subject to temperature drift and the dimensional instability of the ferrite cores. Barometric sensor measurements depend on temperature, weather, and height. As a result, each station pressure measurement needs correction to its equivalent barometric pressure at sea level. Acoustic signals are degraded by noise in the monitoring environment. Sensor efficiency is proportional to its active surface area, which makes larger devices more desirable [17]. Acoustic sensors also have range limits and are considered highly intrusive. PIR sensors work best when motion is within the circumference of the sensors' field of view, and most poorly when the movement is towards the sensor. This makes mounting of PIR sensors on the ceiling a requirement for effective monitoring, because motion is rarely towards the ceiling [15]. Also, heated air degrades PIR-acquired signals and can result in false detections [28]. Pets could trigger false alarms (this is also true for Pressure Mats). Pressure Mats also have limited operational surface area. Switches require a subject to open and close doors/windows and will give a false negative if a subject fails to close a door and the required displacement does not take place [28].

## 3. Distribution of Existing Studies

For the purposes of the work presented in this paper, the sets of ADL from [5–7] have been merged and grouped into categories as shown in Table 1.

**Figure 2.**
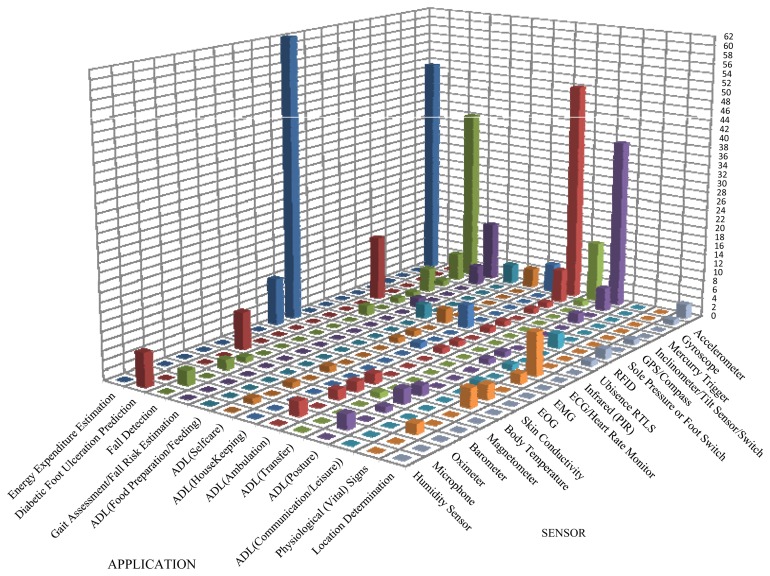
Distribution of research studies based on wearable sensors.

**Figure 3.**
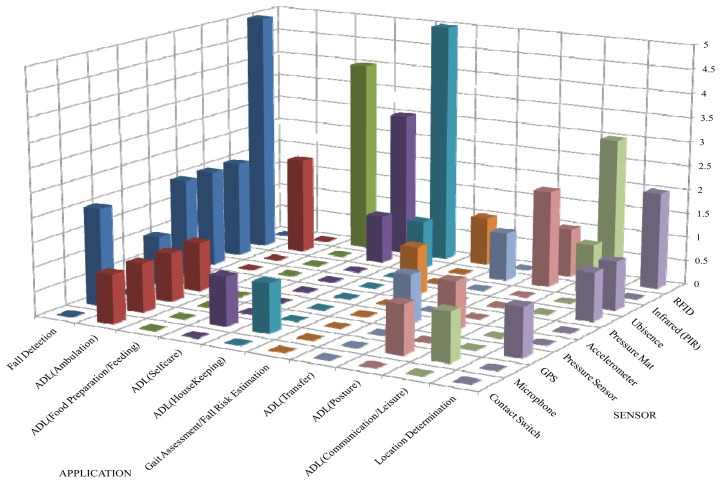
Distribution of research studies based on fixed sensors.

The categories encompass most of the ADL listed by the authors (except shopping, transportation, and handling finances) either as a category or as an element of a category. The set of ADL included in each category are limited to the activities that older adults living independently in their homes and in need of assisted living and rehabilitation could possibly undertake. Categorization of ADL facilitates the articulation of the distribution of existing studies as in Figures 2 and 3, which show graphically the distribution of the reported research studies based on the type of sensor used (wearable or fixed) and the application. The figures indicate that research attention has focused on the different application areas with varying intensities.

A wide range of sensor types (24 different types: twenty used as wearable and nine as fixed sensors) has been used in different research studies. The acquired sensor data have been used for studies in different application areas including gait assessment, fall risk estimation, fall detection, location determination, ADL recognition/classification, physiological (vital) signs assessment for health and wellness evaluation and energy expenditure estimation. Table 2 details the distribution of studies indicating that 88.73% of research across the 13 different application areas (as highlighted in the table) was based on the use of wearable sensors and 11.27% on the use of fixed sensor.

Accelerometers and ECG/Heart Rate Monitors (ECG/HRMs) are the most commonly used wearable sensors. Accelerometers were found applicable in both wearable and fixed capacity and their use spans across 76.92% (10) of the 13 highlighted application areas. The use of ECG/HRM has mostly concentrated in the area of Energy Expenditure Estimation (EEE). EEE is the most studied area followed by ADL (Ambulation), fall detection, and ADL (Posture) with the other areas receiving much less research attention. Some application areas have been totally neglected by research studies, including behaviour trends (patterns) profiling and analysis, affective state detection, and fall context determination. These research gaps are presented in Section 8.

## 4. Sensor Placement and Data Collection

### 4.1. Sensor Placement

Placement locations of monitoring sensors vary based on the type and application. Fixed sensors are generally located within the monitored environment and are most suited for indoor use. For example, the use of PM will entail the placement of a sensor on the floor by the target object (e.g., a toilet seat, a chair, or a doorway). Wearable sensors are placed on in positions on the body that would facilitate the acquisition of data yielding the highest events classification accuracies. Literature highlighted various body placement positions including chest (sternum), forehead, ear lobe, hip (iliac crest), waist (iliac spine), neck, ankle, thigh, wrist, toe, under heel, elbow, knee, shoulder, armpit, sacrum and trunk. Sensors have been placed under the heel and on the toe because those positions play crucial roles in the gait cycle [29]. The authors in [30] placed the monitoring device under the foot to acquire data for classification of the stepping patterns of elderly people and recovering patients. Monitoring devices have also been sewn into garments [31,32]. Different body placement positions result in different signal patterns and classification accuracies [33]. For example, according to [34], incorporating sensors in garments did not prove effective as it resulted in 100% false positives for 155 trial scenarios. Table 3 highlights different placement positions and corresponding classification accuracies from the use of accelerometer data to classify fall, ADL ambulation, posture, and transfer events. The choice of these events and accelerometer for highlighting the impact of placement position on classification accuracy was informed by Figure 2 which indicates that accelerometer is the most common sensors used for studies that addressed these events. The classification values in Table 3 may not necessarily give a completely true picture because the different studies employed different filters for signal pre-processing, extracted different sets of features, and used different data segmentation window lengths and classification approaches. However, the values are based on studies that used accelerometer for monitoring and data acquisition either singly or in fusion with other sensor types.

On the one hand, the waist has been acknowledged as a more effective and acceptable placement position for fall detection. According to [35], “the placement site at the waist has been suggested to be the most efficient since at this site the acceleration signal is similar and evenly distributed between different fall types”. “The origin of the human body model is the waist” [36]. “The placement at the waist is more acceptable from the user point of the view since this option fits well in a belt and it is closer to the centre of gravity of the body” [37]. On the other hand, the authors in [35] found the head to be a better placement position for fall detection applications because the features extracted from head worn sensor data could be used to accurately distinguish between falls and ADL. The data acquired with head worn sensors resulted in highest fall event detection accuracy compared to those acquired with hip and wrist worn sensors [33]. The wrist is not considered an optimal placement position because the measured signals vary widely making it difficult to distinguish between falls and ADL [35]. The wrist is subject to many high acceleration movements that would increase the number of false positives [37]. However, arm worn devices yielded appropriate information for the detection of a set of ADL including cooking, eating, and cleaning [37].

Investigation of optimal sensor placement position in [38] resulted in the following groupings: very low-level activities (e.g., laying down)—wrist and ear; low-level activities (e.g., eating, drinking, reading, and getting dressed)—waist; medium level activities (e.g., walking, vacuuming, and cleaning)—chest and wrist; high level activities (e.g., running and cycling)—ear; and transitional (transfer) activities (e.g., sit-to-stand, laying down-to-stand)—ear. The ideal scenario would be to wear as many sensors as possible on different body positions to track subtle changes in gait and activity in order to improve classification results. This, however, is not practical and minimizing the number of devices worn is of significant practical importance because body worn monitoring device placement raises the issue of wearability and usability.

According to [37], usability strongly affects the effectiveness of the monitoring system. A device worn on the head may afford an excellent impact detection capability but raises high usability concerns. For example according to [39], in a study with waist-mounted monitoring device, users transferred the device from one body location to another due to bruising and discomfort. This implies the need to design wearable monitoring systems that are comfortable to wear and are independent of location and orientation. Also, subjects may take the device off (e.g., to shower) and may not remember or be inclined to wear it, especially at night time (e.g., going to and from the toilet) and, therefore, may not be wearing it when a fall occurs [40].

Also, the wearable monitoring device could be damaged by the impact of a fall, rendering it dysfunctional [39]. In addition, the location of the sensor on the human body relative to the point of impact may modify the “signature” of the signal recorded at the time of the impact [41]. This implies the need for impact proof sensor-based monitoring devices. Furthermore, the device could slip from its placement position and change its calibrated orientation, with respect to the body axis [42]. Calibration, in this context, refers to the alignment of the sensor axis with respect to a reference axis. The axis parallel to the human upper body often constitutes the vertical reference axis. For example, the authors in [42] mounted their monitoring device on the subject's pelvis but did not state the reason for the choice of the alignment angle to the vertical axis, while the researchers in [14] aligned the y-axis of their accelerometer with the reference vertical axis. Orientation of the axes of an accelerometer sensor impacts on the measurements [35]. Calibration is one of the three sources of error in signal acquisition and analysis. The usefulness of acquired data depends on the calibration of the sensor [43].

### 4.2. Data Collection

Data sampling process entails that an analog signal, x(t), is periodically measured every T seconds, such that time is discretized in units of the sampling interval T: *t* = nT, *n* = 0, 1, 2, . . . N, which results in a stream of samples [44]. Essentially, the sampling process represents a chopping operation on the original signal, x(t). Researchers acquired data at a wide range of frequencies for similar measurement scenarios using the same sensor types. For example, for the measurement of human ambulation and ADL scenarios using accelerometers, samples were acquired and recorded at frequencies of 7 Hz [45], 10 Hz [46], and 50 Hz [37], which were deemed to be a good trade-off between saving energy and acquiring enough signal data. Accelerometer signal samples obtained at 10 Hz should be fast enough to capture the necessary amount of data, yet slow enough not to capture unnecessary noise and anomalies [47]. In [48], the authors sampled their accelerometer signals at 50 Hz, while researchers in [33] obtained their accelerometer samples data at the frequency of 200 Hz. Sampling rate set to 120 Hz exceeds the characteristic response of human movement [49]. Other sampling frequencies for accelerometer data include 40 Hz [50], 16 kHz [51], 512, 76.25, 64, and 5 Hz [8,52–54].

The two basic questions are: how best to choose the sampling frequency, *f_s_*, for a measurement scenario; and what is the impact of sampling frequency on classification accuracy? The first question is addressed by sampling theorem [55], which states that for accurate representation of a signal, *x*(*t*), by its time samples, *x*(*nT*), two conditions must be satisfied: *x*(*t*) must be band-limited and *f_s_* must be at least twice the maximum frequency, *f*_max_, in *x*(*t*). That is, *f_s_* = 2*f*_max_ (referred to as the Nyquist rate), is the ideal sampling rate. Essentially, the value of *f*_max_ and hence *T* = 1/*f_s_* depends on the application. “*T* must be small enough so that signal variations that occur between samples are not lost. If *T* is too small there would be too many samples to be processed, if *T* is too large too few samples would be obtained which might lead to the loss of information” [44]. That is, a rapidly varying signal needs sampling at a higher rate and a slowly varying signal needs sampling at a lower rate. According to [34,42], all measured body movements are contained within frequency components below 20 Hz, and in gait 99% of the energy is contained below 15 Hz. Also according to [53], the sampling frequencies can be as low as 10 Hz for posture. As shown in [56], human activity frequencies are between 0 and 20 Hz, and that 98% of the FFT amplitude is contained below 10 Hz. The typical bandwidth of kinematics of normal gait is between 4 and 6 Hz and spectral power analysis from barefoot walking across a force plate shows that 98% of the spectral power is below 10 Hz and over 90% below 5 Hz [57]. In addition, the authors in [58] asserted that it has been demonstrated that 99% of the acceleration power in gait is concentrated below 15 Hz, and that the frequency range of daily activities (performed on a force platform) was shown to be between 0.3 and 3.5 Hz.

**Figure 4.**
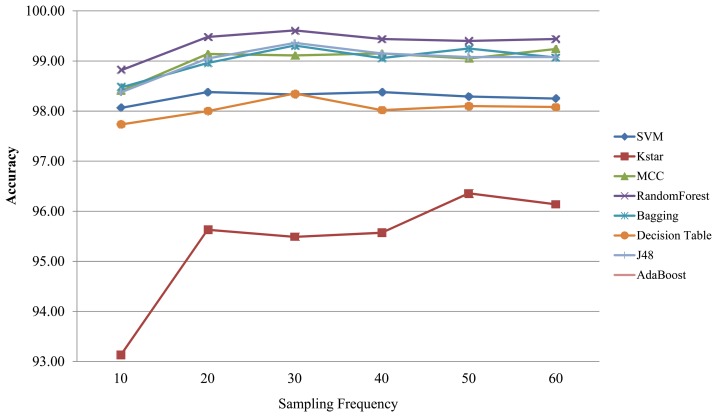
Sampling frequency *vs.* classification accuracy.

To address the second question, we empirically investigated the effects of sampling frequency (*f_s_*) on classification accuracy using a set of six frequencies (10 Hz, 20 Hz, 30 Hz, 40 Hz, 50 Hz, 60 Hz) and a set of 10 machine learning classification algorithms [naïve Bayes, Bagging, Support Vector Machine (SVM), Decision Tree, Kstar, ZeroR, Multi Class Classifier (MCC), J48 (C4.5), AdaBoost, and Random Forest]. It was found that for the eight out of the ten classifiers that yielded meaningful classification accuracies (≥93%) their classification accuracies peaked at the 20 Hz sampling frequency and remained relatively stable without significant increase/decrease with increases in sampling frequency as shown in Figure 4. The lowest classification accuracies from AdaBoost and ZeroR were 65.97 (at the 10 Hz sampling frequency) and 38.93 (at the 30 Hz sampling frequency), respectively.

The results indicate that ADL classification improves with higher sampling rates between 10 Hz and 20 Hz, but only marginally improves or even decays with increases in sampling rates above 20 Hz. Statistically insignificant very weak negative relationship was found between sampling frequency and classification accuracy. Data sampled at 20 Hz is adequate for classifying ADL [59]. The typical *f_s_* values for sampling audio, speech, and biomedical signals are often set at 40 kHz, 8 kHz, and 2 kHz respectively [44].

## 5. Data Processing for Feature Extraction and Selection

**Figure 5.**
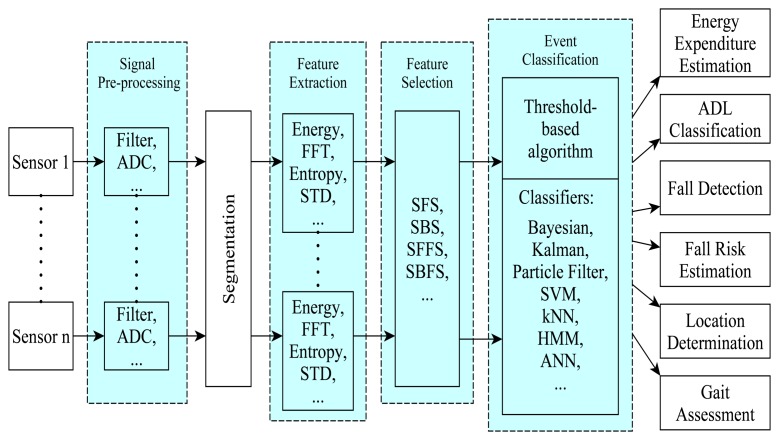
Sensor data processing and analysis (based on [62]).

The sensor data processing and analysis stages include pre-processing (e.g., filtering), segmentation, feature extraction, and feature selection as highlighted in Figure 5. Acquired sensor measurements are often pre-processed to remove noise and artifacts, because signals are easily corrupted by instrumentation noise, random noise, electric and magnetic noise, *etc.* Pre-processing generally entails the use of various filters, which are sometimes arranged into a network. According to [60], noise reduction techniques need to be adapted to the signals and depend on the features to be extracted from the signal. For example, “development of de-noising algorithm for 3D acceleration signals is essential to facilitate accurate assessment of human movement” [61]. According to [61], the typical filters for acceleration signals are median, Butterworth low-pass, discrete wavelet package shrinkage and Kalman filters.

The effects of these filters in terms of signal-to-noise ratio (SNR) and correlation coefficient (R) on 3D accelerometer human mobility and ADL monitoring data were investigated in [61], and the following findings were reported:

1. Kalman filters showed the largest SNR and R values, followed by median filters, discrete wavelet package shrinkage, and then Butterworth low-pass filters.
2. Performance of Butterworth low-pass filters marginally improved over that of Kalman filters after correcting waveform delay for Butterworth low-pass filters.
3. Performance of median filters is related to their window length.
4. Decomposition level influenced real-time performance of discrete wavelet package shrinkage.
5. Filter order and cut-off frequency, if not properly selected, could result in large waveform delay for Butterworth low-pass filters.

A median filter changes the absolute peak value of a signal window and needs to be used cautiously [35]. “In some applications such as falls detection and prevention for elderly people, real-time performance and waveform delay are two important factors that must be considered when developing acceleration filtering algorithms. As a Kalman filter only uses previous data to estimate current state, it has good real-time performance and short delay. A median filter has little waveform delay, but its real-time performance closely relates to its window length N as it needs to wait for about N/2 future data points in order to perform filtering. Wavelet package shrinkage also has little waveform delay, but it needs to wait for at least 2*^J^* future data points to filter noise, *J* refers to decomposition level” [61].

Other pre-processing techniques include Wiener filters (WF), wavelet de-composition (WD), and principal component analysis (PCA) [60]. The use of one or the other depends on the nature of the signal, the statistics of the information, and the noise signal. Sensor data could also be pre-processed to separate the different components of the signal. For example, accelerometer data consist of two components: the linear acceleration component due to body motion (also referred to as the high-frequency, AC, or Body Acceleration (BA) component) and the gravitational acceleration component due to gravity [also referred to as the low-frequency, zero-frequency, DC, or Gravitational Acceleration (GA) component] [63]. Where the data is processed depends on the available resources, application and feature extraction requirements.

### 5.1. Processing Location

A one-way communication often takes place from monitoring device to base station and what is communicated depends on where the acquired data are processed: on-board the monitoring device or on a base station. Some or most of the stages of data processing can be done on-board the device before data and/or processed information are transmitted to the base station. For example, in [46] only pre-processing of data is done on-board before transmission to the base station, while in [10,27,42,64] pre-processing, feature extraction, and fall events detection algorithms are all run on-board the monitoring device. On-board sensor data processing poses challenges due to hardware constraints, which limit the amount of data that can be buffered, the robustness of classification algorithms, and the range of events that can be classified [42]. For instance, ADL classification involves a set of relatively complex algorithms. According to [42], classification of some ADL such as *walking* requires a Fast Fourier Transformation (FFT) of at least 3 seconds of data in order to obtain a magnitude spectrum of the signal. The hardware used in [42] and [34] suffered storage limitations and could not buffer up to 3 s of acquired data on-board. The computational costs, storage requirements, and precision (data representation format) of various feature metrics for accelerometer data in different domains (time, frequency, and discrete) have been quantitatively assessed in [65] in order to determine those that are suitable for on-board implementation. According to [65], the computational cost of a feature extraction algorithm is:

1. *very low* if it requires only a number of operations that have a linear relation to the number of input samples, and the operations are mostly arithmetic additions and subtractions.
2. *low* if it requires a number of operations that are linearly related to the number of input samples, which include multiplications and divisions, and a fixed number of the operations can be advanced arithmetic operations such as square-root or logarithm.
3. *medium* if the metrics include those that are quadratic in terms of the number of input samples, such as simple addition/subtraction or multiplication/division operations and a fixed number of advanced arithmetic operations which can be square-root or logarithm.
4. *high* if the metrics require a number of operations larger than an asymptotic quadratic bound, but where the individual operations are simple arithmetic additions/subtractions or multiplications/ division. This category requires a linear number of advanced operations such as sin or log.

Other issues associated with on-board data processing are energy consumption of the monitoring device and communication bandwidth. A typical sensor-based monitoring device consists basically of a microcontroller, sensor(s), and communication interface(s) (see Figure 1—Memory, Clock and Energy Harvester are optional). According to [42], accelerometers are a major source of power consumption as they are always operational with continuous current supply (even in the idle state as shown in Table 4). The authors in [42] suggested the use of optical-based accelerometers with current rating of 0.4 mA, instead of MEM (micro electromechanical systems) accelerometers with current rating of 4 mA. The available bandwidth limits the data transmission rate.

Table 4 indicates that it is the communication aspect that consumes the highest amount of energy [15]. A Wearable Sensor-based Monitoring Device (WSMD) based on 10 degrees of freedom provided by accelerometer, gyroscope, magnetometer, and barometer was constructed in the course of the study presented in this paper. The WSMD was tested for communication related energy consumption. A current draw of approximately 75 mA was measured with the Bluetooth wireless module attached, when the WSMD was on and had no connection to any other device via any of its communication interfaces (USB and Bluetooth). A current draw of 55 mA (still high compared to value in Table 4) was measured when the device was connected to a smart phone via the Bluetooth interface. The drop in current consumption in communication mode is possibly due to the reduced power consumption of the Bluetooth module when connected and not searching for a device to connect to. Though Bluetooth has the same footprint as ZigBee (also referred to as XBee), ZigBee is better for extending the battery life, but Bluetooth is often used because of its versatility as it facilitates connection to other devices (base stations, smartphones, tablets).

Acquired data are streamed to the base station via a communication channel for storage and processing. A downside is that data can be lost or corrupted during transmission, and the base station is required to be within the communication range. An advantage of processing data on the base station is the abundance of storage and computing resources on the base station [37]. This makes it practically possible to run computationally intensive algorithms such as segmentation, feature extraction and selection, and classification algorithms. Also, the issue of power consumption does not arise and filters of much higher order could be implemented.

Summarily, trade-offs between on-board and base station processing will include power consumption and battery lifespan, storage requirements, and complexity of algorithms. Battery lifespan is a major challenge of long-term monitoring. Subjects could forget to place the device on charge. Therefore, there is a need to implement power saving and renewal techniques on the monitoring hardware (e.g., energy harvesting).

### 5.2. Data Segmentation

Sensor data requires careful segmentation in order to facilitate effective feature extraction. Inappropriate segmentation will result in the extraction of features without discrimination power, which would cause the classification algorithm to yield meaningless results [66]. The segmentation problem is stated as follows: given a time series, *T* (a finite set of samples characterized by time points), partition *T* into segments (windows) of *t* consecutive samples between two time points *a* and *b* that are internally homogeneous with respect to the application. There are different segmentation techniques including Fixed-size Non-overlapping Sliding Window (FNSW), Fixed-size Overlapping Sliding Window (FOSW), Top-Down (ToD), Bottom-UP (BUp), Sliding Window and Bottom-up (SWAB), and Variable-size Sliding Window (VSW) [67–70]. The use of window-based segmentation technique for event classification was criticized because the technique does not facilitate recognition of every single movement/action a subject makes/takes [53]. The set of measurement instances describing an event cannot be classified together, but only a group of instances (a window) can be classified together. The authors in [53] asserted that short duration activities, such as *StandingUp*, cannot be recognized, because window-based segmentation approach requires bigger sample windows for the classification of longer duration activities. Many classification errors in activity recognition are due to poor window size selection [70]. The window could be too short and not cover the span of an activity, or the window could actually overlap two different unrelated activities. The authors also pointed out that a constant sliding window could generate identical features for different activities, which will not improve the classification task. A Variable-size Sliding Window (VSW) segmentation technique aimed at improving classification outcome was proposed in [70]. The VSW is based on the premise that best results could be achieved with a different window size for each activity to be classified, such that the size of each window is large enough to contain the target activity only.

The authors in [68] compared the performance of different segmentation algorithms from a data mining perspective and found that: the FNSW and FOSW segmentation approaches generally produce very poor results; the ToD approach produces reasonable results, but does not scale well; the BUp approach produces excellent results, and scales linearly with the size of the dataset; the SWAB approach scales linearly with the size of the dataset, requires only constant space, and produces high quality approximations of the data. Feature extraction based on FOSW segmentation technique is effective and results in successful outcome [71].

We investigated the effectiveness of window-based segmentation techniques to determine the optimal segmentation approach with respect to the window length and the percentage of adjacent windows overlap for feature extraction resulting in the highest event classification accuracy [72].

**Figure 6.**
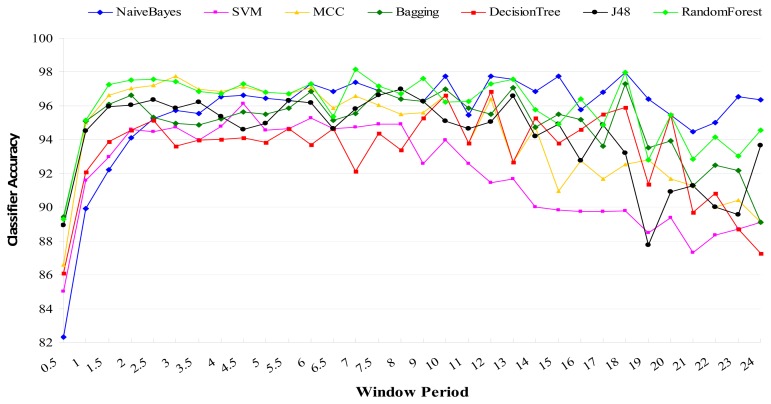
Classification accuracies of NaiveBayes, SVM, MCC, Bagging, Decision Tree, J48, and RandomForest for different window sizes using the FNSW segmentation technique.

**Figure 7.**
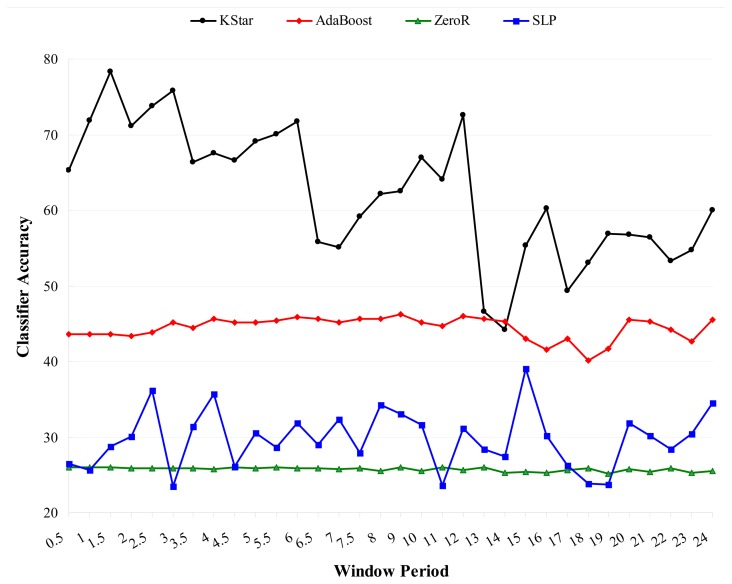
Classification accuracies of Kstar, AdaBoost, ZeroR, and SLP for different window sizes using the FNSW segmentation technique.

**Figure 8.**
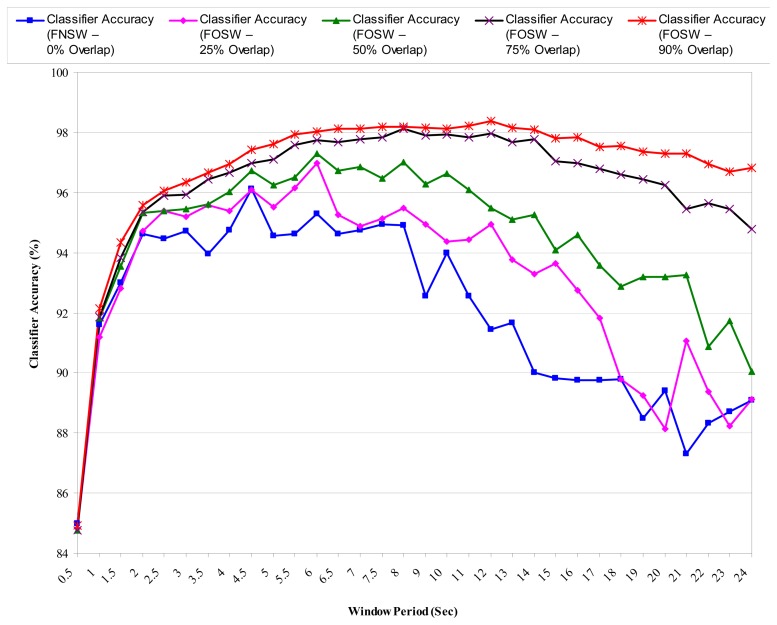
SVM classification accuracies for different window sizes and different window overlaps using SVM classifiers.

It was found that sliding window segmentation techniques are generally effective for human activity classification from sensor data as shown in Figures 6 and 8. Figure 6 shows the classification accuracies for different window sizes for the algorithms with outcomes greater than 82%, while Figure 7 shows outcomes of the same experiments for algorithms with lower performance outcomes.

Figure 8 highlights SVM classification accuracies for different window sizes and different percentage window overlaps, because SVM is the most commonly used classification algorithm (usually because it is the best performing algorithm). The results show that the length (in seconds) of the window impacts significantly on the classification accuracy, and that the strength of the impact (in terms of increases in accuracy) depends on how much (percentage-wise) the windows overlap during segmentation. Table 5 gives the highest accuracies for window overlap size and peak window size combinations when using SVM classifiers.

### 5.3. Feature Extraction

**Figure 9.**
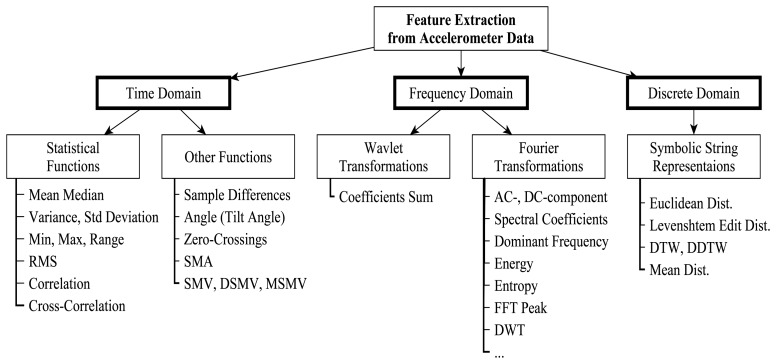
Features from accelerometer data (based on [65]).

The different applications require the extraction of different sets of discriminatory features from the same or different sensor datasets using different metrics. The metric used depends on the required features and representation domain of the dataset (time, frequency, or discrete). Figure 9 highlights different types of features that can be extracted from accelerometer data for different domains of representation.

Analysis of signals in the time domain deals mainly with two dimensions: amplitude and time, which facilitate the extraction of features using statistical metrics including mean, median, variance, standard deviation (STD), minimum (min), maximum (max), range, Root Mean Square (RMS), correlation, and cross-correlation [65]. STD represents the probability distribution of data and is less useful for noisy data, because spurious values can distort results [65]. Other time domain metrics include sample differences, angle (tilt angle), zero-crossings, Signal Magnitude Average (SMA), and Signal Magnitude Vector (SMV). Accelerometer data in the frequency domain have two components: DC and AC. The DC component constitutes the first coefficient in the spectral representation of the signal and its value is often much larger than the remaining spectral coefficients, while the AC component is the dominant frequency component [65]. These components are the basis for the extraction of most of the time and frequency domain features. Accelerometer signal can also be transformed into strings of discrete symbols (discrete domain representation). String represented data can be analyzed for string similarity and pattern discovery using exact or approximate matching and edit distance techniques [65].

In addition, various other metrics have been used for feature extraction from accelerometer data including Shock Response Spectrum (SRS) transform metric from fall vibration signals. The SRS transform [73] likens a fall event to the mass-spring system with respect to the systematic impact of the human body on the floor during a fall event [51]. The SRS is the peak acceleration response of a large number of single degrees of freedom (SDOF) systems each one with a different natural frequency, and it is calculated by convolution integral of the measured signal (input) with each one of the SDOF systems [74]. Full details of the SRS metric and the extraction processes of its temporal features are given in [74]. According to [51], the SRS transform has a total of 133 features and in the very low frequencies their values are close to zero. For example, 93 features of the of the SRS parameter from the frequency bandwidth of 10.1–2,048 Hz of a fall vibration event were extracted and used in [51].

Data acquired with sensors other than accelerometer yield different types of features. For example, temporal features (e.g., sound event length and energy) and spectral features (e.g., Mel Frequency Cepstral Coefficients (MFCC)) are extractable from acoustic sensor data. According to [73], MFCC represent audio signals with frequency bands that are positioned logarithmically and approximate the human auditory system's response more closely than the linearly spaced frequency bands obtained directly from the FFT of the signal. Table 6 highlights features that have been extracted from different sensor data for events classifications and their uses.

For example, the *Mean* metric has been used to recognize the ADLs “sitting” and “standing” [38,79–81], to discriminate between periods of activity and rest [42,82], and as an input to classifiers for multi-activity classification [53,83,84]. *RMS* has been used to distinguish walking patterns [38,42,52] and as an input to classifiers for multi-activity classification [53,85,86]. *SMA* has been used to distinguish between periods of activity and rest in order to identify when a subject is mobilizing and undertaking activities, and when they are immobile [34,42,59,87]. *Energy* and *Entropy* have been used to discriminate between types of ADL [53,88]. *SMV* was used to indicate the degree of movement intensity and as an essential metric in fall detection [34,42]. *STD* has been used for activity classification in [53,81,89].

The authors in [11] compared 14 different metrics for the extraction of features for ambulation events classification based on wavelet transform, time and frequency domain signal characteristics, using accelerometer datasets of ambulation ADL collected from 20 subjects. The accelerometers were worn on three different body positions. They assessed the discrimination power of the features extracted using classification accuracies for each feature set using K-Nearest-Neighbor (KNN) classifier. Their findings showed that although the wavelet transform approach can be used to characterize non-stationary signals, it does not perform as accurately as frequency-based features when classifying dynamic activities. Overall, the best feature sets achieved over 95% inter-subject classification accuracy. The authors suggested that future studies should consider using an FFT feature set derived from an ankle-mounted accelerometer for ambulation ADL classification.

### 5.4. Feature Selection

**Figure 10.**
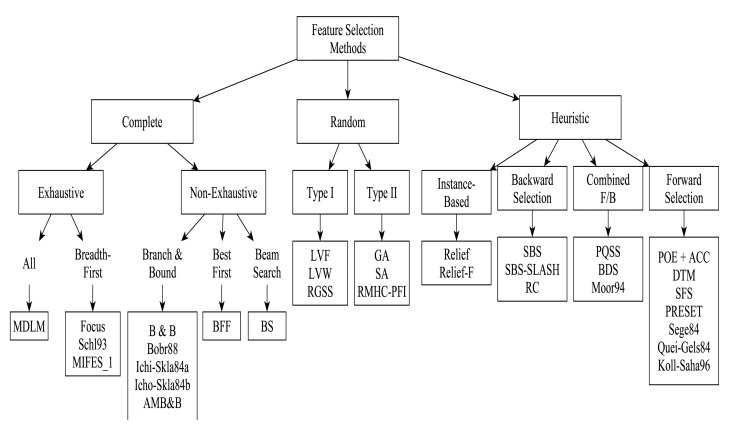
Categories of feature selection algorithms.

The type and number of features required to successfully perform a given classification task depends on the discriminatory qualities of the features. Thus, feature selection is a search problem which finds an optimal subset of *n* features out of the extracted set of *N* features that best discriminate between classes. The aim is to reduce the dimension of the feature vector in order to improve performance and at the same time increase classification accuracy. According to [66], the choice of' features is more important than the selection of a specific classifier because the use of features without discriminant power will degrade classifier performance. There are many different feature selection algorithms which differ according to the criterion function used in searching for good features. An overview of existing feature selection methods in the period from the 1970s to 1997 is presented in [90], identifying the strengths and weaknesses of different methods, while the authors in [91] presented a taxonomy of feature selection techniques and their uses. Commonly used feature selection methods were evaluated in [92], and recommendations were made for which feature selection methods should be used under different research study conditions. A chronological review of literature on feature selection is conducted in [93]. The authors categorized some feature selection algorithms based on the three ways the feature space is searched (complete, heuristic, and random). The results are summarized in Figure 10. The Figure does not give an exhaustive list of feature selection algorithms. For example, Sequential Forward Floating Search (SFFS), Correlation-based Feature Selection (CFS) and K-best algorithms are not listed.

The use of feature selection can improve accuracy [92]. As illustrated in [94], a large number of features can be eliminated without a significant loss of classification performance. The activity classification performance of the following three classification algorithms, Naive Bayes, Hidden Markov Model (HMM) and Viterbi algorithm (a variant of HMM), have a strong relationship with the features used [95]. After evaluating the performance of over ten different feature selection methods, the Sequential Forward Floating Search (SFFS) method was found to be the most powerful algorithm for feature selection [94]. This result was corroborated in [96]. According to [51], the SFFS algorithm [97] has three major steps: inclusion, test, and exclusion. It begins with the inclusion process to select features with the best performance. A test is then conducted on every selected feature in the same iteration to identify the features that will reduce the overall performance of the algorithm. If such a feature exists, SFFS would then exclude this feature from further cycles. The iterations are continued until all features have been examined. SFFS was used to rank the discriminatory powers of a set of 110 features from accelerometer and acoustic sensor data (that is, vibration and sound signals) for fall detection application and the algorithm chose a set of 17 top performing features which yielded classification performance of 97.5% sensitivity and 98.6% specificity [51]. The authors in [96] found the worst performing feature selection algorithm to be K-best. Correlation-based Feature Selection (CFS) is another feature selection technique commonly used by researchers [98,99]. It is inbuilt in WEKA is based on the assumption that discriminatory features should be highly correlated with the given class, but uncorrelated with each other [12].

## 6. Applications

There are different application areas as highlighted in the introduction including gait assessment, fall risk estimation, fall detection, ADL recognition/classification, energy expenditure estimation, diabetic foot ulceration prediction, physiological (vital) signs, and location determination. Research studies employed a wide range of classification algorithms (such as threshold-based and machine learning) and sensors. The most commonly used classification algorithms include Support Vector Machine (SVM), K-Nearest Neighbour (KNN), C4.5 (implemented as J48 in WEKA), Artificial Neural Networks (ANN), Dynamic Bayesian Networks (DBN), Bayesian Networks, Hidden Markov Models (HMM), and Gauss Markov Models (GMM), among others. Figure 2 indicates that fall detection, ADL (ambulation, transfer, and posture) classification are the most studied application areas in literature.

Table 7 highlights some classification accuracies in literature for these four most studied applications and events based on different classification approaches.

### 6.1. Fall Detection

Figures 2 and 3 indicate that accelerometer (in both fixed and wearable capacity), inclinometer/Tilt sensor/switch, and PIR sensors are the most commonly used sensors for fall detection applications. Other less used sensors include gyroscope, pressure mat, Ubisense RTLS, and microphone (in both fixed and wearable capacity), barometer and magnetometer. The precision of Ubisence RTLS with respect to the tag position estimation is not considered high [100]. Accelerometer is superior in its ability to yield multiple features that facilitate high event classification accuracy.

There is no standard fall detection technique or algorithm. Researchers generally implement and use their own proprietary threshold-based algorithms or machine learning classification algorithms among others. Literature highlighted eight main characteristic attributes of fall events highlighted in Table 8. Each attribute yields parameters and features assessable from different sensor data.

Fall detection is generally centered on these characteristic attributes, but any one fall event may not necessarily exhibit all of them. For example, a hard impact on a fall surface may cause sharp peaks in acceleration magnitude and high amplitude floor vibrations that can be sensed. Soft impacts may not be easily sensed [37,51]. Body impacts with the fall surface may or may not generate detectable sounds [51].

The Signal Magnitude Area (SMA), calculated according to Equation (1), is used to derive a measure of a subject's level of activity in order to distinguish between periods of activity and inactivity (rest) [42]:

(1) $$\begin{array}{ll} SMA & =\frac{1}{n}(\int_{0}^{n}|x(t)|dt+\int_{0}^{n}|y(t)|dt+\int_{0}^{n}|z(t)|dt)\\ & =\frac{1}{n}\overset{n}{\underset{j=0}{\text{∑}}}(|x_{j}|+|y_{j}|+|z_{j}|) \end{array}$$

where *n* is the total number of samples in a sample data window and *x_j_*, *y_j_*, and *z_j_* represent the *j*^th^ data sample in the sample data window for each of the accelerometer axes. It is evaluated from the BM component of the accelerometer signal. Test for post-fall inactivity is based on the premise that for “serious” falls, subjects will most likely remain immobilized in posture and place [41]. A subject is deemed inactive if the SMA value is less than a given estimated threshold value. The literature, however, has not highlighted any benchmark SMA threshold values. That is, researchers determine and use their own proprietary values, often without stating the value used.

*Acceleration peak* or movement intensity is the most common feature for fall detection using upper and/or lower acceleration threshold values. The assumption is that most fall events are characterized by impact on the fall surface causing sharp peaks in acceleration magnitude [37]. The Signal Magnitude Vector (SMV), also referred to as Sum Vector (SV), is used to evaluate the degree of movement intensity and is the most commonly used parameter for deriving acceleration peak thresholds. The feature is generally estimated from the BM component of accelerometer signal using the Signal Magnitude Vector (SMV) or Sum Vector (SV) metric in Equation (2):

(2) $$SMV=\sqrt{x_{i}^{2}+y_{i}^{2}+z_{i}^{2}}$$

where *x_j_*, *y_j_*, and *z_j_* represent the *j*^th^ data sample in a window of the BM component of the accelerometer data. Table 9 highlights some SMV threshold values that have been used in different studies, the sensor placement position for data collection, and classification accuracies achieved.

A challenge for peak acceleration threshold-based fall detection approach is the calculation of the optimal threshold value(s) that will yield the highest classification accuracy. Currently, there are no standard values, as Table 9 indicates. It is worth noting that if the threshold value is too high, the system may miss some fall events (sensitivity < 100%), but it will never generate false positives (specificity = 100%). If the threshold value is too low, the system will successfully detect all fall events (sensitivity = 100%), but may generate some false positives (specificity < 100%) [37]. The peak acceleration value depends on the placement position of the wearable sensor-based monitoring device on the subject's body (e.g., wrist, hip, ankle, *etc.*) [35]. The accuracy is also affected by the fact that there are ADL characterized by acceleration peaks similar to those of fall events. The authors found that the type of the filter used for pre-processing the data could impact on the peak acceleration value (e.g., median filter changes the absolute peak value of the signal). The literature indicated that different fall scenarios yield different peak accelerations. There is a need to investigate the optimal value bearing in mind that there are activity events characterized by acceleration peaks similar to those of real fall events.

The pose (posture) of a static object is its position (orientation relative to a frame of reference) and attitude (inclination or tilt) [104]. Fall events can be classified on the basis of (post-fall) postural orientation and rotational speed of the trunk [34]. According to [15], “strictly related to a fall is the posture, which can be determined by monitoring the tilt transition of the trunk and legs and the angular coordinates”. Body tilt angle is generally taken to be the angle between the positive z-axis (the axis parallel to a human's upper body) and the gravitational vector, *g*, of accelerometer GF component.

According to [42], “if tilt angle is 0 to 60 degrees, it is classified as upright, whereas values of 60 to 120 indicate a lying posture; any greater a tilt angle and the user is classified as inverted”. *Change in postural height* (altitude) is evaluated from pre-processed barometric sensor data the target being to assess altitude changes per data window of *n* samples [34,105]. The metric in Equation (3) is used to evaluate changes in postural height:

(3) $$\Delta H_{i}=\frac{1}{n}[(\overset{k=j+(n-1)}{\underset{k=j}{\text{∑}}}h_{k})-(\overset{k=j-1}{\underset{k=j-n}{\text{∑}}}h_{k})]$$

where *h_k_* is the *k^th^* data sample in a converted barometric signal window, *n* is the window size, and *j* is the window index. Essentially, *ΔH_i_* gives a measure of the altitude trend calculated by subtracting the average altitude of the previous corrected barometric data window from that of the current window.

Fall events are also classified on the basis of *changes in orientation* of the body trunk [34], referred to as angular displacement [106]. Gyroscope measures the rate of change of orientation in 3D and the derivative of the angular position over time for each coordinate is assessed as shown in Equation (4):

(4) $$\begin{array}{l} \theta_{x}^{\prime }=d\theta_{x}/dt\\ \theta_{x}(t)=\int_{0}^{t}\theta_{x}^{\prime }(t)dt\approx \overset{t}{\underset{0}{\text{∑}}}\theta_{x}^{\prime }(t)T \end{array}$$

$\overset{t}{\underset{0}{\text{∑}}}\theta_{x}^{\prime }(t)T$ is an approximation which takes the sum of a finite number of samples in the interval *T*, the sampling period. The issue with this approximation is that if the gyroscope data change faster than the sampling frequency (drift), errors will be introduced, which may not be detected, and the integral approximation will be incorrect [106]. Drift, which is a major problem of gyroscopes, increases with time.

According to [46], the *direction of a fall* can be determined from pre-processed accelerometer data by evaluating the azimuth angle *φ* (the angle between the x-axis and a line from the origin to the data point projected on the same plane as the reference direction) for the *k*^th^ data sample as in Equation (5). The assumption is that the z-axis of the sensor is aligned with the subject's body vertical axis.

(5) $$\varphi =\arctan2(y_{k}/x_{k})$$

Fall events have also been classified on the basis of spectral features from fall surface *vibration* and *sound* data. This is based on the assertion that for a fall event, the body impact with a fall surface generates detectable sound and repeated shock (vibration) signals. That is, the repeated impacts of a body part with the fall surface generate vibrations that are likened to a mass-spring system which are transmitted throughout the fall surface [51]. According to [27]:

1. The vibration signature of a floor impact generated by a human fall is significantly different from that generated by normal daily activities like walking and tapping.
2. The vibration signature of a floor impact generated by a human fall is significantly different from that generated by objects falling on the floor.
3. Different floor surface types (for example, a concrete floor covered with linoleum and a concrete slab floor) produce different amplitudes in the vibration signals at equivalent distances from the sensor, requiring different detection thresholds for different floor types.
4. Different floor types have different vibration ranges for detection (for instance, 5 m for concrete slab floors, and 7 m for mezzanine concrete floor covered with linoleum).

These differences in the response of different floor types to different excitation activities have been exploited for fall detection [27]. Accelerometer taped to the floor in a monitored environment has been used to measure fall vibration patterns [51]. An acoustic sensor also taped to the floor was used to measure fall sound signals. The issue is that “hard” fall events cause high-amplitude floor vibrations that can be easily sensed, while “soft” fall events may not be as easily sensed. Also, there are different kinds of vibration sounds from human motion that can vary in strength and duration depending on the type of motion event, such as short events (e.g., steps) and long events (e.g., falls) [51]. In view of these, the authors in [51] suggested that if a surface (e.g., a floor) produces high amplitude vibrations >10 g RMS at the measurement point, a relatively low sensitivity (10 mV/g) sensor may be preferable, but if the vibration is <10 g RMS, a high sensitivity (100 mV/g) sensor may be more appropriate. Furthermore, the performance was not significantly affected by the different floor treatments tested (linoleum, carpet, and carpet with foam padding). It was also found that the sensor-based monitoring devices should be placed no closer than 1.5 m to walls on mezzanine concrete floor, and no closer than 1.2 m on concrete slab floor to minimize false alarms from any fall event in a neighboring apartment [27]. The features that are most commonly extracted from vibration and sound signals for fall classification, as detailed in [51], are summarized in Table 10.

These features have been used to achieve fall event classification accuracies of 100% sensitivity with 100% specificity, and 97.7% sensitivity with 98.6% specificity in [27] and [51], respectively. The main issue with this fall event classification approach is that acoustic sensors are considered highly intrusive, raising privacy concerns among monitored subjects.

### 6.2. Gait Assessment

Traditionally, there are two methods of undertaking gait measurement: automated and manual. Automated motion laboratories with highly accurate computer-based force and optical tracking sensors (e.g., OptoTrack), instrumented walkways (e.g., GAITRite system), piezodynamometric platforms, and instrumented floors are used to analyze the motion of body segments in order to measure various characteristic parameters, such as stride length, stride frequency, and instantaneous walking speed [76]. Though these systems produce well-quantified and accurate results, they are often restricted to clinical laboratory settings due to size and cost considerations, and have limited applications. Manual measurement involves a series of tests including Timed “Up and Go” (TUG), functional reach tests, and visual observations to assess the gait of monitored subjects [76,107]. TUG is a simple mobility test during which the subject is asked to stand up from a chair with an armrest, to walk for a distance of 3 m, to turn around, to walk back, and to sit down [108]. The time required to complete the test is measured. If it is below 20 s, the subject is considered to have no mobility issues, and if it is above 30 s, the subject is taken to have serious mobility challenges. Times between 20 s and 30 s require further assessment [108]. The clinical score STRATIFY (see [109,110] for details) is also used to derive measures of gait parameters. These manual methods are inexpensive, but the results are qualitative, unreliable, and difficult to compare across multiple measurement [76]. Moreover, the scores are intended for hospital use and have to be assessed by experienced medical professionals and, therefore, may not be suitable for home-based monitoring [108].

Currently, sensor-based approaches to gait assessment are being increasingly adopted as they facilitates quantitative and repeatable analysis over extended time periods [76]. Gait analysis using wearable sensors is an inexpensive, convenient, and efficient way of providing useful information for multiple health-related applications and it shows great potential [3]. Gait assessment using wearable sensors could enhance the comprehension of gait strategies [111]. The authors in [112] used measurement data from a footswitch to quantify temporal and distance aspects of gait, while data from “GaitShoe”, a sensor suite consisting of accelerometer, gyroscope, Force Sensitive Resistor (FSR), Polyvinylidine Flouride stripe, bend sensor, and electric field sensor, were used for quantitative gait analysis [76]. Other studies have also used these different sensors for gait assessment. These include estimation of temporal characteristics of gait using data from body-worn accelerometers and inside-footwear pressure sensor [57,113,114], and quantification of the differences between shuffling and walking using force-sensitive resistor (FSR) measurements of pressure distribution beneath the foot [115,116]. Patterns in gait were also analyzed using data from two FSRs positioned under the heel and in the general area under the toes [117,118]. During gait most body motion occurs in the lower limbs, so most studies attached the sensors on the thigh, shank, ankle, foot, heel, and toe of the subjects. Essentially, the sensor data measurements yield parameters and features that characterize human locomotion, such as velocity, cadence, stride length, heel-strike timing, toe-off timing, Planthar flexion, gait phases, and orientation. Table 11 highlights parameters/features extracted from the GaitShoe sensors data in [76].

**Figure 11.**
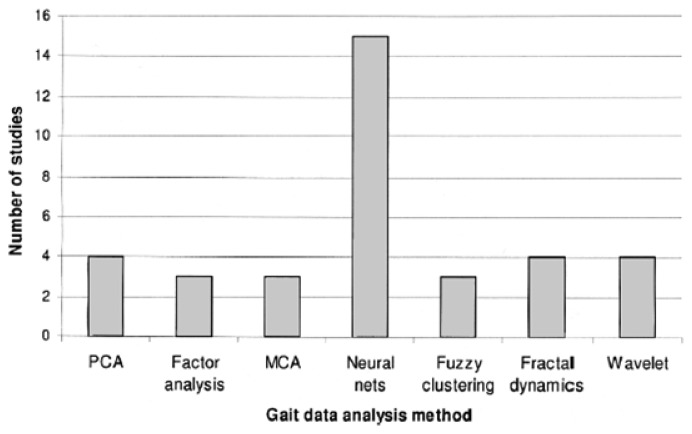
Algorithmic techniques for gait classification.

Algorithmic techniques for gait classification include Artificial Neural Networks (ANN) and Principal Component Analysis (PCA), among others. Pattern recognition algorithms were used to define gait cycle transitions for triggering functional electrical stimulation (FES) in patients with incomplete spinal injury [119,120]. However, neural nets seem to be the most commonly used and efficient. Out of a set 36 studies, 42% (*n* = 15) used neural nets (see Figure 11) [121]. The authors in [122] used four different algorithms (neural nets, fuzzy inference, self-organizing map, and neuro-fuzzy algorithms) with the same set of data features from EMG sensor worn on a lower limb, and found neural nets to be the most effective for gait classification. The accuracies for these approaches were 94%, 56%, 91.4%, and 76% respectively.

### 6.3. Fall Risk Estimation

One third of falls by elderly adults involve environmental hazards in the home, the most common being stumbling or tripping over objects [4]. Environment related fall risk factors are generally detectable by physical observations, while physiological related fall risk factors are traditionally assessed with clinical instruments and scores (such as STRATIFY, Tinetti, geriatric team score and TUG), which identify fall risks and their gait related assessable parameters. The current trend is to assess these parameters from sensor-based motion measurements data. The use of wearable inertial sensors was investigated in [101] to provide objective data for fall risks estimation and to compare the predictive performance of sensor-based methods with conventional and established clinical methods. The authors found that among clinical instruments and scores for fall risk assessment, the geriatric team score outperforms STRATIFY and TUG, while the sensor-based model was able to identify more persons at risk of fall than the clinical instruments and scores. The authors concluded that sensor-based measurements with wearable device may contribute significant information to conventional methods and are feasible in unsupervised settings.

Sensor-based measurements have been used to distinguish between high and low fall risk subjects based on gait parameters and changes in trunk posture [123]. The gait-related parameters of the fall risk factors identified by the STRATIFY score have been extracted from sensor data measured during TUG tests [108]. Walking patterns have been assessed from foot switch and mercury trigger sensor data in order to predict future falls [30]. Data acquired with FSRs positioned under the heel and toes has been used to find patterns in gait for fall risk assessment [117,118,124]. Several types of wearable sensors are suitable for the acquisition of mobility data for gait and fall risk assessment including accelerometers, gyroscope, force resistive sensors, inclinometers, goniometers, magneto-resistive and Electromyography (EMG) sensors, each of which can give various characteristic measures of the human gait and phases [108,125]. It was concluded that a single tri-axial accelerometer worn on the trunk at the level of the pelvis was the most suitable because it is small, unobtrusive, has low power consumption and delivers well-interpretable data [108,126]. A single inertial sensor was suitable for identifying stride, step, and stance duration, and it provides the opportunity to measure other gait parameters outside of the traditional laboratory [102]. Figure 2 indicates that the most commonly used sensors for gait assessment and fall risk estimation are accelerometer, gyroscope, and sole pressure or foot switch.

Although the analysis of gait stability may allow the identification of subjects at risk, the definition of gait stability is still fuzzy with many direct and indirect measures for quantification of gait being proposed in literature [103]. Measures of trunk accelerations are crucial in the assessment of gait stability for risk estimation as the trunk segment is known to play a critical role in regulating gait-related oscillations in all directions [103]. According to [103], falls in older adults often occur during walking, and the trunk position is known to play a critical role in the balance control. Therefore, the analysis of trunk kinematics during gait could present a more viable approach to fall risk estimation based on such parameters as harmonic ratio (HR), index of harmonicity (IH), multiscale entropy (MSE), and recurrence quantification analysis (RQA) of trunk accelerations. Their parameters are not dependent on step detection (the metrics for these features are given in [103]). The authors in [103] investigated the association between these parameters and fall history, and found that univariate associations with fall history for MSE and RQA parameters in the AP direction gave the best classification results. MSE and RQA were found to be positively associated with fall history and could, hence, represent useful tools in the identification of subjects for fall prevention programs [103].

The latest results in fall risk estimation are presented in [127] and focus on the dynamic imaging of human footprints based on fibre-optic sensors embedded in the carpet. This work showed “the capability of such imaging technology to study variations in gait and walking patterns, as well as the footprint of a human body lying in various positions” [127]. According to [108], “a wearing position close to the body's center of mass might be reasonable to measure gait and may also be accepted by older people: an unwieldy wearing position or multiple sensors should be avoided for a future everyday use”.

### 6.4. ADL Classification

ADL classification uses data from a wide range of monitoring sensors and classification algorithms. The category and level of granularity of an ADL impact on the choice of sensors for its monitoring. For example according to [24], activities characterized by the manipulation of objects during their performance can be recognized from sensor data about object touches (object-based activity classification). RFID is well suited for the monitoring of object-based activities whereby the RFID reader is fitted in the subject's hand glove, while the tags are positioned on the target objects that the subject uses (touches). RFID antennae are able to discriminate among specific instances of objects that are otherwise the same (e.g., two different spoons) with 0% false positive rate [24]. The *Selfcare and HouseKeeping* ADL categories are mostly object-based as they are disambiguated by key objects and, therefore, could be monitored with RFID. Fusion of RFID and accelerometer data facilitates the recognition of an activity and the subject's posture while undertaking the activity. Examples include subject standing while ironing, standing while brushing teeth, sitting while reading [77]. The ADL “eating” and “drinking” (while sitting at the table) were classified in [128] using data from wrist worn accelerometer and RFID (the reader was installed under the table surface). However, RFID-based monitoring is tag intensive. For instance, over 14 tags were deployed in [129].

**Figure 12.**
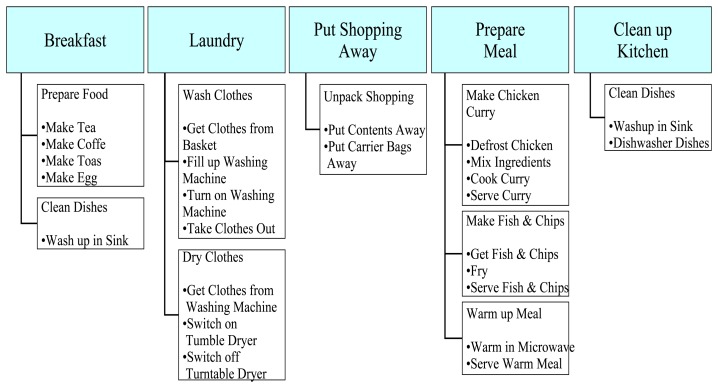
Hierarchical abstraction of ADL for recognition/classification.

Some of the elements of an ADL category can be broken down into constituent sub-ADLs, while a sub-ADL can further be broken down to Atomic ADL (tasks) in order to facilitate recognition and classification. According to [7], “complexity varies widely within each level, so that specific activities can be arranged in the hierarchy only with knowledge of both the within- and among-levels complexity of the activity. A functioning subject may thus be assessed by measuring instruments designed to tap into representative behaviour at each level and within the range of competences appropriate to the individual”. That is, an ADL can be abstracted into hierarchies with the highest level of abstraction referred to as the goal and the lowest level of abstraction consisting of task events (as demonstrated in Figure 12) and recognition of an ADL can be based on recognizing its constituent task events [129]. The number of levels of sub-goals between a task and its associated goals depends on the complexity of the goal.

Classification of ADL *ambulation*, *posture*, and *transfer* relies heavily on the extraction and selection of appropriate features from sensor data and often entails distinguishing between periods of activity and inactivity. Accelerometer data is deemed the most suitable for these events since during inactivity only the GA component is recorded, while both the GA and BA components are recorded during activity. Figure 2 indicates that accelerometer is the most common wearable sensor used for the classification of these ADL with the waist and hip being the most common placement positions.

*Ambulation ADL* (mobility) classification aims to differentiate different walking patterns and types (walking on level ground and walking up/down a stairway). According to [130], “while walking, acceleration oscillates, and the peak amplitude varies according to the road condition, but the peak interval remains constant”. Walking patterns can be differentiated based on acceleration signals in the vertical and anteroposterior directions [52].

According to [52], “Level walking has three peaks in a walking cycle, and stairway walking has one or two peaks”. Table 7 indicates that up to 99.9% accuracy has been achieved for ADL classification using fuzzy logic classification technique. The *posture* ADL has been classified with 100% accuracy using threshold-based algorithms. *Posture ADL*, standing and sitting, have been successfully distinguished from accelerometer data using the SMV and DSMV features from accelerometer data [80]. Accelerometer data give relatively high error rate when differentiating between standing and sitting, because the angle of tilt from the vertical axis varies by a non-significant margin between the two ADL and is particularly sensitive to the placement of the device [39]. Also, the data acquired with gyroscopes have been proven more accurate for distinguishing between sitting and standing. Furthermore, differentiating between lying due to a fall and lying as an ADL event is challenging [39]. This is where the assertion that fall events exhibit high acceleration peaks is supposed to be useful. However, not all fall events are characterized by an impact [46].

Using a set of feature metrics and three feature selection algorithms, the authors in [38] sought to provide answers to the following two questions: what is the ideal sensor placement location for a given group of activities; and which time and frequency domain features in wearable accelerometer data are most relevant for discriminating different activity types? The authors found that the features listed in Table 12 (not necessary in any particular order) proved the most effective for differentiating between the ADL ambulation, posture, and transfer. According to [38], averaged entropy over three axes was highly ranked by all the three feature selection algorithms used for the investigation, especially the one extracted from an ankle worn sensor. Averaged mean of cross covariance between each two axes) was also highly ranked, especially for the one extracted from ear and chest worn sensor data. Frequency features, particularly the energy of 0.2 Hz window around the main frequency divided by the total FFT energy, were also ranked highly for the knee, ankle, and ear worn sensors, as they reflect repetitive walking patterns [38].

Figure 2 shows that the few studies that addressed the ADL *communication* and *Leisure* used RFID and wrist worn accelerometer [76,77], EOG [26] and ECG/Heart Rate Monitor. RFID based classification is generally based on object touch. Assessment from EOG data was based on the analysis of repetitive eye movement patterns by estimating eye movement and position parameters, saccade, fixation, and blink based on the features that were extracted and selected (see Table 13). According to [26], the EOG features highlighted in Table 10 constitute a representative set for the classification of much broader range of communication and leisure activities in daily life including such activities as copying a text, reading a printed paper, taking hand-written notes, watching video, browsing the web, and periods of no specific activity.

### 6.5. Energy Expenditure Estimation

**Figure 13.**
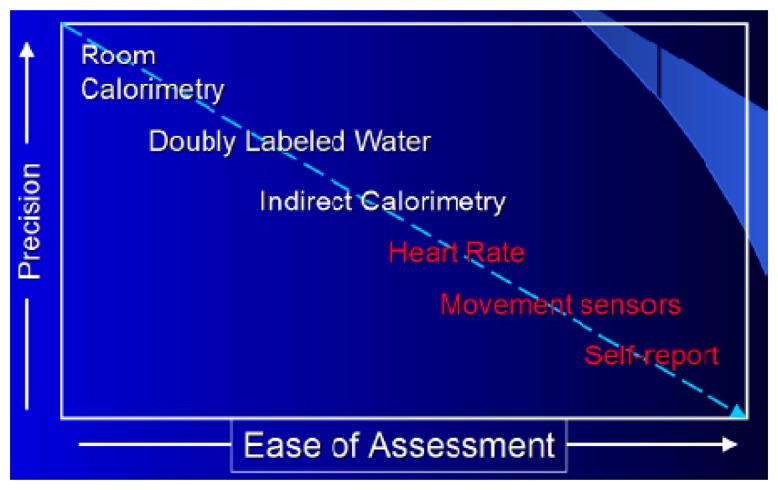
EE estimation tools.

Activities of daily living are often associated with Energy Expenditure (EE) but according to [131] “the energy cost of physical activity may not necessarily be equivalent to body movement”. The validity and reliability of energy expenditure estimates have been established in [132]. Different methods of estimating EE by a subject include room calorimetry, doubly labeled water, indirect calorimetry, heart rate, inertial sensors, and self-report. The authors in [133,134] reviewed the different tools for measuring physical activity and total energy expenditure and addressed the advantages and limitations of the tools. The level of precision and ease of EE assessment with these methods are shown in Figure 13. The figure indicates that the room calorimetry method has the highest precision, but is not easy to use, while inertial sensors and self-report are the easiest to use, but do not give high precision.

The self-report method (the traditional instrument of choice especially when large populations are to be assessed) uses questionnaires, activity diaries, and recall interviews, and is somewhat limited in objectivity. “Supplementing questionnaires with a personal interview does elicit more detailed data, but activity monitors provide a more objective measure of activity that can be used as an adjunct to questionnaires” [133]. “Doubly-labeled water is considered the gold standard to measure energy expenditure over time. It is a method of indirect calorimetry, in which carbon dioxide production is tracked from metabolism of specific isotopes in the labeled water. The technique is expensive and requires specific expertise” [132]. Objective activity monitors have been increasingly used to overcome the limitations of self-report and the high precision measures. “Several models of activity monitors are capable of collecting and storing data for many days, weeks, or even months. More importantly, the internal real-time clocks of these monitors allow the discrimination of activity patterns” [133]. Figure 2 indicates that ECG sensors, heart rate monitor, accelerometer, and EMG are the most commonly used sensors for EE estimation studies. According to [131], there is a “linear relationship between heart rate and energy expenditure during steady state work load with large muscle groups”. Heart rate monitors are inexpensive, but energy expenditure estimates can be confounded by other factors that increase heart rate such as caffeine or stress [132].

“Accelerometer data, commonly expressed as the dimensionless unit, “counts,” are inherently neither meaningful nor interpretable” [135]. The accelerometric counts need to be translated into quantitative estimates of caloric expenditure. “In general, the approach to translate accelerometer counts into energy expenditure has been to compare activity counts and oxygen consumption measured during performance of a series of activities that reflect activities of daily living” [135]. “After the simultaneous counts and oxygen consumption are obtained, regression methods are applied to determine the relationship between the two measures, and an equation to predict energy expenditure from accelerometer counts and/or a count threshold for a particular intensity of activity is determined. In most studies, a single linear regression is the analytic approach” [135]. For example, two-regression approach was used to estimate energy expenditure from accelerometer data [78]. The choice of regression was based on the observed Coefficient of Variation (CV) among 10 second accelerometer counts. According to [135], this approach helps to address the disparities in the relationship between counts and energy expenditure related to activity type. “Regular rhythmic accelerations as observed in walking and running result in a low CV that leads to the choice of one regression equation, whereas more variable activities with a resulting larger CV lead to use of the other equation. The combination of the estimates from the two regressions results in more accurate and more precise energy expenditure estimates for the activities examined. The utilization of the CV to determine regression selection is an appropriate and unique response to the problem of discriminating locomotion from more mixed movement activities” [135].

There is a strong association (*r* > 0.75) between EE and activity accelerometer measurement counts in controlled settings. EE prediction equations and activity intensity levels differ depending on the calibration activities performed and the settings of these activities. Prediction equations established in the laboratory are not valid for free-living activity EE estimation. The authors in [136] compared accelerometer-based EE estimation algorithms and platforms, and found high correlations between the accelerometer-regressed EE estimates and the reference dataset. Assessing agreement between EE estimation from accelerometer data and from self-reports showed that the magnitude of the association was not significantly affected by age or weight status, but was significantly higher in males than in females [137]. Also, self-report underestimated activity EE in overweight/obese and older adolescents. The authors in [137] concluded that assessment of activity EE is complex and may require a combination of methods. Future studies should combine the use of sensor-based monitors with suitable questionnaires [133]. According to [138], the use of accelerometers along with questionnaires may yield more reliable and accurate measurements. There are existing off-the-shelf products, such as SenseWear WMS armband, that combine accelerometry with other physiologic measures (e.g., heart rate, galvanic skin response). According to [58], there was a large variability in accelerometer output and their validity to assess daily physical activity. So far, there is little evidence that adding other physiological measures, such as heart rate, significantly improves the estimation of energy expenditure.

The EE of an activity may be underestimated depending upon the placement of the sensor-based monitoring device [132]. EE studies have been done with multiple accelerometers worn on a number of body placement positions including the trunk and extremities. The waist or hip is the most common sensor placement position for EE estimation [132,135–138]. According to [132], positioning of monitors is a potential source of error in studies, as it was found that a uniaxial accelerometer yielded significantly different results depending on which of three parts of the hip it was worn on. Also, wearing the monitor on the right versus the left hip is an issue of consideration as wearing a monitor on different hips generated significantly different activity measurements. Having a snug fit between the monitor and the subject has been recommended to limit extraneous movement [132].

Also, “accelerometers can be used to approximate energy expenditure, however, they do not capture the full energy cost of certain activities, such as walking while carrying a load or walking uphill, because acceleration patterns do not change under these conditions” [132]. According to [135], waist-mounted accelerometers cannot accurately detect upper body movements or the effort related to lifting or carrying loads.

### 6.6. Diabetic Foot Ulceration Prediction, Physiological (Vital) Signs, and Location Determination

The application areas of diabetic foot ulceration prediction, physiological (vital) signs, and location determination seem to be increasingly gaining research attention. Diabetic patients are said to be at risk of lower extremity skin breakdown, foot deformities, and imbalances, which could cause repeated high pressures and pain under the forefoot during ambulation. These pressures and pain have temperature and moisture (humidity) implications inside the shoe [139,140]. Research is focused on the use of in-shoe sensors (accelerometer, sole pressure and foot switch—see Figure 2) to measure plantar pressures inside the shoe during walking and ADL by patients with diabetes mellitus and peripheral neuropathy [141,142]. For example, an insole-based electronic system can monitor temperature, pressure, and humidity, storing the data in a battery-powered device [139,143]. The pressure sensors were located at the heel and under three metatarsal heads. Temperature sensors were located under the medial metatarsal head and under the heel. The humidity sensor was located in the toe of the shoe. The data was used to quantify the conditions inside shoes in order to predict the progression of skin breakdown and ulceration. A battery powered sensor-based device was used to monitor peak plantar pressures during gait and successfully identified “steps” that indicated peak plantar pressure for an extended period of time [144]. The authors in [142–145] also used in-sole pressure sensors to measure plantar pressure during activities of daily living by diabetic patients. It was found that pressures during other activities were not always well predicted by walking pressures, and measurement during level walking alone cannot be considered to fully define the plantar pressure affecting a foot in a particular shoe during ADL [142].

Physiological (vital) signs monitoring studies used ECG/HRM, EMG, body temperature, skin conductivity sensors and oximeters. A feature extraction algorithm for ECG signal is presented in [66] that is based on string representation of the signal. The string representation is then analyzed to extract the optimal feature set that would facilitate ECG recognition. Morphological changes in the shape of the waves of the ECG signal become visible signs of an illness of the heart muscle, and in almost all diagnosis, recognition is based on wave analysis. This includes amplitude and durations of the QRS complex, the P and T waves, the ST-T deformation, the P-P and R-R intervals. Evaluating these parameters requires that the onsets and ends of the waves are fixed [66]. Other studies that detailed feature extraction from ECG data include [146–148].

## 7. Research Gaps and Possible Future Research Directions

Behaviour Trend (pattern) profiling and analysis from monitoring sensor data is an area of study that has been neglected. Trend analysis would facilitate the detection of behavioural deviations and anomalies that could indicate health related issues. Short-term monitoring aids the generation of labeled datasets, which facilitates event classification. Long-term monitoring sensor data may be more suitable for trend profiling and analysis for the detection of unusual (atypical) behavioural events. However, long-term monitoring poses a number of challenges including data labeling, which raise the issue of profiling and analysis with integrity. Short term monitoring, in this context, is within designated finite time period, which could range from seconds to days, mainly for the purposes of evaluation study of the event(s), while long-term monitoring involves recording measurements for weeks to years, which provide a trend. “Short-term monitoring also provides essential information for interpreting long-term monitoring studies” [149].

Affective States detection is another area that seems to have been overlooked. Affective states (e.g., emotions of pain, happiness, sadness, anger, fear, disgust, and, surprise, intents, desires, and moods), which comprise both conscious and unconscious reactions, can be detected in both verbal and non-verbal expressions [150]. Affective states can be indicated by vocal features (e.g., speech rate, intonation, or pitch) and body/facial gestures (e.g., eye movements, facial expressions, head gestures, or body gestures). Affective states can reveal information regarding the psychological and physiological state of a subject in addition to context-related cues and non-verbal displays. Affective states can be causes of behaviour and, thus, can be used to explain and predict subjects' behaviour. For example according to [150], combined measurements of heart rate variability and speech analysis could give insights into a subject's emotions. Three speech variables pitch, intensity, and energy could enable discrimination of different emotions and arousal. Also, galvanic skin response and heart rate sensor data could provide a good indication of stress levels. This indicates that the monitoring of human physiological parameters could contribute significantly to behaviour trend analysis. According to [151], the best approaches for inferring affective states from non-verbal expressions in speech include: creation of a general framework that can handle a variety of affective states and their expressions rather than a system that is specific to predefined emotions; recognition of affective states that often occur in everyday life (rather than strong expressions of basic emotions that are rarely experienced or seen); and handling of various affective states that occur simultaneously in a speaker-independent manner.

Fall context determination is another area that demands research attention. Context has been defined by [152] as “any information that can be used to characterize the situation of an entity (person, place, or object)”. For example, fall context could be the vector of pieces of information that characterize the circumstance within which a fall event occurred, subject's pre-fall activity, and the post-fall state. Most existing fall detection techniques are focused on the detection of isolated fall events under clearly defined conditions and generation of a notification signal in the form of an alarm. Thus, they provide little or no information about the event itself, the event(s) leading to it, and subject status after the event. These pieces of information could prove vital for post-fall subject management, prevention of future falls, and improvement of fall detection strategies. “Context information can play a significant role in improving fall detection accuracy” [153].

Furthermore, more research efforts are required in the areas of Gait Assessment/Fall Risk Estimation, ADL(FoodPreparation/Feeding), and ADL(Selfcare) recognition and classification. These are vital for independent living by elderly adults, but they were considered in only 21 (4.3%), 11 (2.25%), and 17 (3.48%), respectively, out of the 488 research studies across 13 different application areas. Falls are preventable fall risks are estimated. Possible causes of falls are referred to as fall risk factors [4]. Fall risk factors for elderly adults include, in addition to environmental hazards, medication and physiological factors, most of which precipitate gait variability. Hence, fall risks could be detected through posture, physiological (vital) signs, visual, and gait assessment. No single risk factor causes all falls, but the greater the number of risk factors to which a subject is exposed, the greater the probability of a fall [4]. Estimation of fall risk factors is more desirable than a retrospective classification of the specific precipitating causes, because by identifying the risk factors early appropriate preventive strategies can be devised and instituted [154].

Also, the perceived advantages of fixed sensor based monitoring over wearable sensor based monitoring is that the issues of wearability, damage from fall impact, and integrity of the acquired data (as it may be difficult to determine if the device is being worn or is being manipulated), do not arise. It is therefore imperative that future research focus is geared more towards fixed sensor based monitoring based monitoring using a wider range of non-intrusive fixed sensors. For example, the use of ubisense RTLS ADL (Transfer) in gait assessment/fall risk estimation is yet to be explored. A system that could sense and classify the activities of multiple subjects simultaneously is also desirable.

## 8. Challenges and Ethical Issues

### 8.1. Challenges

#### 8.1.1. Data Collection

The most common data collection method by researchers is the use of healthy adults to simulate different fall and ADL scenarios in controlled laboratory environments. Falls are simulated onto floors with cushions, such as mattresses and sponge mats [42,46], and on hard surfaces and crash mats with participants wearing protective gear to avoid injuries [37,49,155]. Falls have also been simulated using dummies, mannequins, and human-sized dolls [27,51]. Some studies have elicited the participation of elderly adults (mostly healthy elderly adults not needing monitoring) living independently in their homes to simulate ADL scenarios [108,156–0]. Simulation tasks are often initiated independently and voluntarily or based on prompts, with scenarios repeated a number of times.

These simulated falls and ADL do not necessarily represent real events, and the falls being performed on cushioned floors alter the characteristics of the fall impact [39]. It is not clear to what extent the classification procedures and results correspond to real fall and ADL events by elderly adults in need of monitoring. Also, most of the studies are based on short-term monitoring because long-term monitoring poses challenges. In addition, eliciting the participation of a set of real subjects (elderly adults who actually require monitoring and living independently in their homes) is a significant challenge. Where subjects are available, ethical approval has to be obtained. There are also issues of subjects' understanding and willingness. Participants may not always have deep understanding of required simulation outcomes and inadvertently vary their execution of the same ambulation and ADL (mixing steps, forgetting steps, executing the task faster or slower than required) [159]. This results in data without integrity. Also in real life, subjects tend to interleave two or more ADL (e.g., tidying and washing up while cooking). Different ADL could share common sub-tasks (e.g., “cookMeal” and “makeHotDrink” may both require using the kettle to boil water). Moreover, activities can be interrupted (e.g., phone call can interrupt the preparation of a meal), which would postpone the current task or cause it to be forgotten, or both activities could be done in parallel. In addition, an elderly adult could be sharing a house with other people or pets. This will most likely introduce “noise” in the sensor data. In view of these, it was suggested in [160] that monitoring systems need to implement flexibility and adaptability to address such issues.

Research needs to identify and address the mitigating factors for studies with real subjects (elderly adults living independently in their homes and in need of monitoring) so that outcomes are applicable in real-life.

#### 8.1.2. Trial Scenarios

Different studies are based on different sets of fall and ADL trial scenarios, which highlights the lack of a standard set of experimental scenarios for research studies. This complicates comparisons of the outcomes of different studies, because common procedures were not utilized to carry out the trials, nor were common criteria adopted for their evaluation. For example, it may be meaningless to compare 100% detection rate for an algorithm tested on only three fall scenarios and seven ADL, simulated by one subject, to an algorithm tested on 20 fall scenarios and 32 ADL, simulated by six subjects, with a detection rate of 68% [39].

The authors in [15,41] proposed sets of fall and ADL trial scenarios as possible steps towards standardization, but failed to give reasons for their choices of the suggested scenarios. We propose the adoption of the ADL listed in Table 2 as the standard set of ADL trial scenarios, because that set encompasses most of the ADL originally proposed in [5,7]. Also, the set of ADL are limited to the activities that older adults living independently in their homes and requiring monitoring (due to age, health, or mobility issues) could possibly undertake independently. In addition, most of the ADL have been evaluated for their usefulness as physiological mechanisms in a variety of institutions and facilities serving community-resident older people. According to [7], “the outcome of the evaluation studies offer evidence that validate the index [of independence] as true measure of primary biological and psychosocial functions”. The behaviours measured by the ADL are described in details in [5,7].

There is a need to detect every fall event. It is therefore imperative that fall event detection studies encompass as many fall trial scenarios as possible. Basically, most falls could be in any one of the three main directions: forward—often due to stumbling/tripping; backward—often due to slipping; and sideways—often due to loss of balance. The subject could end up in any one of the six positions: supine, prone, fowler's, lateral and crumbled. According to [161], 82% of falls by elderly adults occur from standing height, while the authors in [162] established that falls by the elderly adults occur mostly during ambulation (walking). Studies have also shown that forward fall while walking is the most common type of fall by elderly adults. 60% of falls in elderly adults happen in the forward direction. Researchers may, therefore, want to focus more attention on the forward fall scenarios.

#### 8.1.3. General Lack of Confidence

There is a general lack of confidence in the use of existing monitoring systems in real environments with real subjects [163]. For example, it is difficult to determine if the monitoring device is being worn or is being manually manipulated by the subject [34]. According to [164], subject compliance with respect to wearing monitoring device is an important issue as some wear the device for the required duration while some don't. The authors in [164] investigated participants' compliance with wearing monitoring devices and found that subjects wore the device on average for 13.9 h in a 24-hour day with older people having 0.5% more wearing hours. Also, current monitoring approaches do not offer decisive information for determining whether an activity has or has not actually been performed, and therefore there are no assurances or guarantees of the outcomes produced by the system. A two-step activity recognition method was suggested in [59] as a way to address the issue. The first step is to recognize an activity, and the second step is to recognize the effect of the activity as a verification of the actual occurrence of the activity. The authors concluded that experimental results demonstrate that activity verification increases accuracy consistently for each classified activity. The research community may want to explore means of activity verification.

#### 8.1.4. Distinguishing between Fall and ADL Events

Distinguishing between fall and ADL events still poses challenges, though each event has distinct characteristic signatures in the sensor data. According to [47], fall events will show clear acceleration peaks making it possible to differentiate fall events from ADL events. Acceleration peaks of ADL do not usually exceed 3 g, but fall events usually range several g's higher, suggesting that the difference in acceleration peaks can be utilized for distinguishing fall events from ADL events [165,166]. In addition, postural orientation is one of the key differentiating factors between fall and ADL events [167]. However, there are activity events (such as lying down quickly, sitting down quickly, running, and jumping) that generate fall-like acceleration peaks [15]. A subject turning quite vigorously between lying postures could also generate peaks similar to those of a fall event [42]. Also, turning sharply while standing could pose challenges to fall detection as pointed out in [168], due to confusion between standing and sharp turning.

### 8.2. Ethical Issues

Ethical issues relate to standards of conduct, types of devices used (in terms of technological capabilities), and the way the devices are used as components to support subjects in their own homes [169]. Ethical considerations are imperative for all application areas. According to [169], ethical issues can be considered under four mutually nonexclusive categories: respect for autonomy (the right of each subject to control his/her own life and freedom from undesired interference); privacy (monitoring devices compromise users' privacy in different ways and to different extents, depending on the sensor, with video camera considered to be the most intrusive); benefit; and use of resources.

Some ethical issues to be considered with respect to the use of wearable monitoring devices as listed in [169] are given Table 14.

## 9. Conclusions

A wide range of sensors have been used for human ambulation, activity, and physiological (vital) signs sensing. Accelerometer is the most commonly used sensor for a number of reasons including low cost, low error, and high accuracy. However, it has been shown that combining accelerometer data with data from other sensors (sensor fusion) provides a more robust and reliable system. Additional sensors provide a way to acquire more useful data to enhance system performance. Sensor location for data acquisition is an important factor which determines the quality of acquired signals and impacts on system performance (e.g., event classification accuracies). There is no standard set or fixed number of features that can be extracted from any sensor data, which seems to be especially the case for accelerometer data.

The performance measures used in most of the reviewed literature are accuracy, sensitivity and specificity (based on the confusion matrix). The problem with accuracy is that it assumes equal cost for all error types and gives only the base-rate value (that is, it predicts the predominant class) such that 70% accuracy = 30% error. That is, if accuracy changes from 70% to 95% (36% increase in accuracy), for example, error would reduce from 30% to 5%. Also, the value of accuracy is subjective depending on the problem and crosslinks between the factors involved in the problem solution. Researchers may wish to consider the use of other performance measures including weighted (cost-sensitive) accuracy, F-measure, ROC, Mitre F-Score, Kappa score, Balanced Accuracy, Log-loss and Brier score. ROC is considered a better statistical foundation than most other measures and is taken as a standard measure in medicine and biology.

Significant amount of research studies is still required in the subject area in order to address the related challenges and limitations discussed in this paper and to arrive at a system that is universally applicable. Furthermore, the constructive convergence of research directions is required in many aspects. The research community needs to define and adopt a common set of definitions, standards, protocols, algorithms, and systems evaluation techniques. This will facilitate a meaningful comparison of research results and the buildup of data pool for meta-analyses. Also, there is a need to ensure that wearable monitoring devices are fit for purpose both in terms of effectiveness and wearability.

## Figures and Tables

**Table 1.** Categories of ADL.

**Category**	**ADL**
Food Preparation	cookMeal, micorwaveMeal, roastMeal, makeHotDrink, makeColdDrink
Feeding	eating, drinking
Selfcare	bathing, dressing, grooming, toileting, medicating
HouseKeeping	tidying, vacuuming, laundry, cleaning, washingUp
Ambulation	walkingLevel, walkingUpStaircase, walkingDownStaircase
Transfer	sit-to-stand, stand-to-sit, lie-to-sit, lie-to-stand, stand-to-lie, sit-to-lie
Posture	lying, sitting, standing, tripod
Communication	makingPhoneCall, receivingPhoneCall
Leisure	reading, watchingTV

**Table 2.** Distribution of existing research.

**S/No**	**Application Area**	**Number of STUDIES**	**Number of Studies Based on WSMD**	**Number of Studies Based on FSMD**
1	Gait Assessment/Fall Risk Estimation	21	19	2
2	Fall Detection	74	60	14
3	Location Determination	13	8	5
4	ADL (Food Preparation/Feeding) classification	11	7	4
5	ADL (Selfcare) classification	17	12	5
6	ADL (Housekeeping) classification	19	12	7
7	ADL (Ambulation) classification	76	70	6
8	ADL (Transfer) classification	17	15	2
9	ADL (Posture) classification	60	55	5
10	ADL (Communication/Leisure) classification	10	5	5
11	Physiological (Vital) Signs Assessment	20	20	0
12	Energy Expenditure Estimation	121	121	0
13	Diabetic Foot Ulceration Prediction	29	29	0

	**TOTAL**	**488**	**433**	**55**

**Table 3.** Placement positions *vs.* accuracy.

**Sensor Placement Position**	**Classification Accuracy (%)**			

	**Fall Detection**	**ADL Ambulation**	**ADL Posture**	**ADL Transfer**
Waist/Hip	100.00	98.00	94.60	94.10
Wrist/Watch	75.00	90.00	92.86	97.10
Chest	99.10	95.90	95.50	-
Trunk/Torso	100.00	100.00	100.00	100.00
Head	100.00	100.00	100.00	100.00
Ankle	93.30	89.71	94.60	16.70
Thigh	95.00	95.10	94.60	84.30
Arm	97.20	89.71	95.67	-
Shoulder	-	90.00	94.60	-
Knee	-	85.00	90.00	-
Armpit	94.00	-	97.96	-
In pocket	-	95.00	99.70	-
Cell phone	98.00	92.90	94.80	70.00

**Table 4.** Accelerometer current consumption.

**State of the Accelerometer-Based Monitoring Device**	**Current Consumption (mA)**
Idle state	7.91
Sampling + ADC + update of variables	8.31
Transmitting data	29.8

**Table 5.** The highest accuracies for window overlap and window size combinations using SVM classifiers.

**Sliding Window Segmentation Approach**	**Window Overlap Value (%)**	**Window Size**	**Highest Classification Accuracy (%)**
No Overlap	0	4.5	96.12
Overlap	25	6	96.97
Overlap	50	6	97.30
Overlap	75	8	98.13
Overlap	90	12	98.38

**Table 6.** Classified event, parameters/features and data source.

**Classified Event**	**Sensor Data**	**Parameters/Features**	**Classification Approach**	**Accuracy**
**Fall Detection**	Accelerometer, Barometer[34] Accelerometer, Barometer [46] Accelerometer [42] Microphone [75] Accelerometer (as a vibration or impact sensor) [27]	SMV, SMA, Tilt angle, Differential pressure Magnitude of a moving-window standard deviation, Standard deviation of the vector magnitude, ratio of the polar angle calculated in consecutive windows of 20 samples, difference in the values of the polar angle in consecutive windows SMA, SMV, Orientation, Mel Freq. Cepstral Coefficients (MFCC), Sound event length, Sound event energy, Steered response power (SRP) (A12) Vibration event length, Vibration event energy, Shock response spectrum,	Threshold-based algorithm Not stated Threshold-based algorithm kNN Not stated	1. 96.9% 2. 94.12% 3. 95.6% 4. 95% 5. 100% Sensitivity, And 100% Specificity
**Gait Assessment and Fall Risk Estimation**	GaitShoe [76]: accelerometer, bend sensor, gyroscope, Force Sensitive Resistor (FSR), Polyvinylidine Flouride stripe, and electric field sensor.	Stride length, Stride velocity (integration of acceleration); orientation; force distribution under foot, heel-strike timing, and toe-off; heel-strike timing, toe-off timing; Plantar flexion/dorsi-flexion, Flexion at metatarsals; Height of foot above ground	Not stated	Not stated
**ADL Food Preparation&Feeding**	RFID [24]	Object touch	Not stated	81.2%
**ADL Selfcare**	RFID [24] Accelerometers, RFID [77]	Object touch Acceleration, Object touch	Not stated Proprietary algorithm	1. 81.2% 2. 92.95%
**ADL House Keeping**	RFID [24] Accelerometers, RFID [77]	Object touch Acceleration, Object touch	Not stated Proprietary algorithm	1. 81.2% 2. 100%
**ADL (Ambulation, Transfer, Posture)**	Accelerometer [38]	Averaged variance over three axes, RMS of signal derivative, mean of signal derivative, average entropy over three axes, average cross correlation between each two axes, average range over three axes, average main frequency of the FFT over three axes, total signal energy averaged over three axes, energy of 0.2 window around the main frequency over total FFT energy (three axes average), Averaged skewness over three axes, Averaged Kurtosis over three axes, Averaged range of cross covariance between each two axes, Averaged mean of cross covariance between each two axes.	k-Nearest Neighbour (kNN, *k* = 1 − 5, 7); BN with Gaussian priors	Not stated
**ADL Communication and Leisure**	1. EOG [26] 2. Accelerometers, RFID [77]	1. Sacade (mean, variance, max amplitude, *etc.*), Saccade duration, fixation (mean, variance, amplitude, *etc.*), Fixation (time between each two saccades) duration, Average blink rate blink (mean, variance, max amplitude, *etc.*). 2. Acceleration, Object touch	1. SVM 2. Proprietary algorithm	1. precision of 76.1% and recall of 70.5% 2. 93.02%
**Energy Expenditure**	Accelerometer [78]	Coefficient of Variation (CV) for six 10s epochs within a 1min period, Vo_2_, average CV and the average counts per minute were calculated for minutes 4–9 of each activity	Two-regression model	95%
**Location Determination**		Orientation, tilt angle,		
**Subject Active or Inactive)**	1. Accelerometer 2. Accelerometer [42]	1. Mean, SMA, Variance, STD. 2. SMA	1. Not stated 2. Threshold-based algorithm	1. 100% 2. 100%
**Movement or Activity Intensity**	Accelerometer [42,65]	Sample differences, Integral of RMS, Mean of Minmax, SMV, Cross correlation		
**Checking and Comparing Signals**	Accelerometer [65]	Correlation coefficients, Sample differences, Signal Correlation, Cross correlation, Dynamic time warping		

**Table 7.** Accuracies of common classification approaches for the four most studied applications.

**Classification Approach**	**Classification Accuracy (%)**			

	**Fall Detection**	**ADL Ambulation**	**ADL Transfer**	**ADL Posture**
SVM	92.30	93.00	68.00	99.63
C4.5/J48		89.71	93.80	97.10
Naïve Bayes	97.30	84.00	93.30	94.60
Decision trees	80.00	90.80		
ANN		95.00	89.30	
KNN	84.44	90.00	78.70	90.00
DBN	80.00	98.00		98.00
BN	93.00	94.00	91.30	99.00
HMM	82.00	87.05		
Fuzzy Logic	80.00	99.90	97.00	98.70
GMM	41.00	91.30		
Threshold-based algorithms	100.00	100.00	100.00	100.00
Ensembles		90.00		90.00

**Table 8.** Fall characteristic attributes and sensor data source.

**Characteristic Attribute**	**Sensor Data for Extraction of Relevant Parameters/Features**
Inactivity (post-fall period of inactivity)	Accelerometer (wearable)
Acceleration peak (high movement intensity) from impact with the fall surface	Accelerometer (wearable)
Rotation of body trunk	Gyroscope (wearable)
Change in body position (orientation and tilt angle)	Accelerometer, Magnetometer
Change in postural height or altitude	Barometer (wearable)
Vibration from impact with the fall surface	Accelerometer (fixed)
Sound from impact with the fall surface	Microphone (wearable or fixed)
Direction of fall (backward, forward, *etc.*) which could reveal its cause	Accelerometer (wearable)

**Table 9.** Fall characteristic attributes and sensor data source.

**SMV Threshold Value (g)**	**Fall detection Accuracy (%)**	**Sensor Placement Position**	**Reference**
1.8	95.6	Waist	[32]
1.8	-	Waist	[101]
2	100	Waist	[35]
3.5	-	-	[102]
3.52	100 (specificity)	Trunk	[103]
2.74	83.33 (specificity)	Thigh	[103]
3	100 (sensitivity)	Waist	[37]
6.5	41/100 (sensitivity/specificity)	Wrist	[35]
1.7	100	Head	[35]
3.09	99.1	Chest	[32]
3.35	97.9	Under arm	[32]
1.8	96.9	Waist	[34]

**Table 10.** Fall vibration and sound features.

**Category**	**Parameter per Signal Window**	**Possible Number of Extractable Features**	**Source Signal**
Temporal parameters	Vibration event length	1	Vibration
	Vibration event energy	1	Vibration
	Sound event length	1	Sound
	Sound event energy	1	Sound
Spectral parameters	SRS	93	Vibration
	MFCC	13	Sound

**Table 11.** Parameters/features extracted from the GaitShoe sensors data.

**Sensor**	**Parameters/Features**	**Sensor Output**
Accelerometer	Stride length and stride velocity, and other velocities and displacements	Voltage change corresponding to acceleration: single integration of acceleration yields velocity, double integration yields distance (integration done after correcting for gravitational component)
Gyroscope	Orientation	Voltage change corresponding to angular velocity: single integration yields angle of rotation
Force sensitive resistors	Force distribution under foot and heel strike timing, toe-off timing	Resistance change corresponding to applied force across the sensor, resulting from change in compression of the sensor
Polyvinylidene fluoride stripe	Heel strike timing and toe-off timing	Voltage change corresponding to dynamic pressure across the sensor
Bend sensor	Planthar flexion/dorsi-flexion, flexion at metatarsals	Resistance change corresponding to flexion angle, resulting from strain on the sensor
Electric field sensor	Height of foot above floor	Capacitance corresponding to distance

**Table 12.** Optimal features for ambulation, posture and transfer ADL classification.

**Feature number**	**Description**
1	Averaged variance over three axes
2	RMS of signal derivative
3	Mean of signal derivative
4	Average entropy over three axes
5	Average cross correlation between two axes
6	Average range over three axes
7	Average main frequency of the FFT over three axes
8	Total signal energy averaged over three axes
9	Energy of 0.2 Hz window around the main frequency over the total FFT energy (averaged over three axes)
10	Averaged skew over three axes
11	Averaged Kurtosis over three axes
12	Averaged range of cross co-variation between two axes
13	Averaged mean of cross co-variance between two axes

**Table 13.** Selected optimal features for the classification of the ADL communication and leisure from EOG data.

**Parameter**	**Features**
Saccade	Mean, variance, and maximum of signal amplitude or rate of small or large positive or negative saccades in horizontal or vertical direction
Fixation	Mean and variance of horizontal and vertical signal amplitude within a duration of a fixation or rate of fixations
Blink	Mean and variance of the blink duration or blink rate
Workbook	Workbook size, maximum and the difference between maximum and minimum, mean, variance of all occurrences in the workbook

**Table 14.** Ethical issues.

**Category**	**Relevant Issues**
Anatomy	1. Does the subject have the capacity to consent to or refuse their use and all aspects of their use?
	2. Has the subject been fully informed of possible effects of their use, of who has access to the information and what the responses will be?
	3. Are there mechanisms in place to ensure continuing consent?
	4. Does the subject have control over the use of and responses to the device?
	5. If the subject lacks capacity to consent, is the consent procedure appropriate?
Privacy	1. In what ways do the uses of and responses to the device invade the subject's privacy?
	2. How can such invasion be minimized?
	3. Do the benefits of using the device outweigh the invasion of privacy?
Benefit	1. What are the expected benefits of using the device both in the short and longer term?
	2. What are the dangers and possible unwanted effects of their use both in the short and long term?
	3. How can the benefits be maximized and the unwanted effects minimized?
	4. Where do the overall best interests lie?

## References

[1] UN (United Nations) (2011). World Population to reach 10 billion by 2100 if Fertility in all Countries Converges to Replacement Level.

[2] Sutherland D.H., Kaufman K.R., Moitoza J.R., Willis D.J. (1994). Kinematics of Normal Human Walking. Human Locomotion.

[3] Tao W., Liu T., Zheng R., Feng H. (2012). Gait analysis using wearable sensors. Sensors.

[4] Tremblay K.R., Barber C.E. (2012). Preventing Falls in the Elderly.

[5] Katz S., Ford A., Moskowitz R., Jackson B. (1963). The index of ADL: A standardized measure of biological and psychosocial function. J. Am. Med. Assoc..

[6] Lowenthal M.J. (1964). Lives in Distress: The Paths of the Elderly to the Psychiatric Ward.

[7] Lawton M.P., Brody E.M. (1969). Assessment of older people: Self-maintaining and instrumental activities of daily living Instrumental Activities of Daily Living Scale (ADL). Gerontologist.

[8] Huynh D.T.G. (2008). Human Activity Recognition with Wearable Sensors. Ph.D. Thesis.

[9] Hattersley J. Energy Expenditure and Accelerometers..

[10] Hijaz F., Afzal N., Ahmad T., Hasan O. (2010). Survey of fall detection and daily activity monitoring techniques. Electr. Eng..

[11] Preece S., Goulermas J., Kenney L., Howard D. (2009). Activity identification using body-mounted sensors—A review of classification techniques. Physiol. Meas..

[12] Lara O.D., Labrador M.A. A Survey on Human Activity Recognition Using Wearable Sensors..

[13] Yang C., Hsu Y. (2010). A review of accelerometry-based wearable motion detectors for physical activity monitoring. Sensors.

[14] Perry J.T., Kellog S., Vaidya S.M., Youn J. Survey and Evaluation of Real-Time Fall Detection Approaches.

[15] Abbate S., Avvenuti M., Corsini P., Vecchio A. (2010). Monitoring of Human Movements for Fall Detection and Activities Recognition in Elderly Care Using Wireless Sensor Network: A Survey. Wireless Sensor Networks: Application-Centric Design.

[16] Khaleghi B., Khamis A., Karray F.O., Razavi S.N. (2013). Multisensor data fusion: A review of the state-of-the-art. Inf. Fusion.

[17] Zhou H., Hu H. (2008). Human motion tracking for rehabilitation—A survey. J. Biomed. Signal Proc. Control.

[18] (2008). Capsil Wiki. Video Monitoring-Based Fall Detectors..

[19] Zhang C., Tian Y., Capezuti E. Privacy Preserving Automatic Fall Detection for Elderly Using RGBD Cameras.

[20] MRT (Microsoft Research Team) (2010). Windows Kinect SDK Beta.

[21] Zhang H., Parker L.E. 4-Dimensional Local Spatio-Temporal Features for Human Activity Recognition.

[22] Li W., Zhang Z., Liu Z. Action Recognition based on a Bag of 3D Points.

[23] Sung J., Ponce C., Selman B., Saxena A. Human Activity Detection from RGBD Images.

[24] Patterson D.J., Fox D., Kautz H., Philipose M. Fine-Grained Activity Recognition by Aggregating Abstract Object Usage.

[25] Philipose M., Fishkin K.P., Perkowitz M. (2004). Inferring activities from interactions with objects. IEEE Pervasive Comput..

[26] Bulling A., Ward J.A., Gellersen H., Troster G. (2011). Eye movement analysis for activity recognition using electrooculography. IEEE. Trans. Patt. Anal. Mach. Int..

[27] Alwan M., Rajendran P., Kell S., Mack D. (2006). A Smart and passive floor-vibration based fall detector for elderly. Inf. Commun. Technol..

[28] Noury N., Herve T., Rialle V., Virone G., Mercier E., Morey G., Moro A., Porcheron T. Monitoring Behavior in Home Using a Smart Fall Sensor and Position Sensors.

[29] Willis D.J. (2000). Ambulation Monitoring Fall Detection System Using Dynamic Belief Networks. Ph.D. Thesis.

[30] Nicholson A.E. (1996). A case study in dynamic belief networks: Monitoring walking, fall prediction and detection. Lect. Notes Comput. Sci..

[31] Bourke A.K., van de Ven P.W.J., Chaya A.E. The Design and Development of a Long-Term Fall Detection System Incorporated into a Custom Vest for the Elderly.

[32] Bourke A.K., van de Ven P.W.J., Chaya A.E. Testing of a Long-Term Fall Detection System Incorporated into a Custom Vest for the Elderly.

[33] Lindemann U., Hock U. (2005). Evaluation of a fall detector based on accelerometers: A pilot study. Med. Biol. Eng. Comput..

[34] Bianchi F., Redmond S.J., Narayanan M.R. (2010). Barometric pressure and tri-axial accelerometry-based falls event detection. IEEE. Trans. Neural Syst. Reh. Eng..

[35] Kangas M., Kontilla A., Winblad I., Jamsa T. Determination of Simple Thresholds for Accelerometry-based Parameters for Fall Detection.

[36] Bachmann E.R. (2000). Inertial and Magnetic Tracking of Limb Segment Orientation for Inserting Humans into Synthetic Environments. Ph.D. Thesis.

[37] Abbate S., Avvenuti M., Cola G., Corsini P. Recognition of False Alarms in Fall Detection.

[38] Atallah L., Lo B., King R., Yang G. (2010). Sensor placement for activity detection using wearable accelerometers. IEEE Trans. Biomed. Circuits Syst..

[39] Shany T., Redmond S.J., Narayanan M.R., Lovell N.H. (2011). Sensors-based wearable systems for monitoring of human movement and falls. IEEE Sens. J..

[40] Ariani A., Redmond S.J., Chang D., Lovell N.H. Software Simulation of Unobtrusive Falls Detection at Night-Time Using Passive Infrared and Pressure Mat Sensors.

[41] Noury N., Fleury A., Rumeau P. Fall detection—Principles and Methods.

[42] Karantonis D.M., Narayanan M.R., Mathie M., Lovell N.H. (2006). Implementation of a real-time human movement classifier using a triaxial accelerometer for ambulatory monitoring. IEEE Trans. Inf. Technol. Biomed..

[43] Dargie W. (2006). A Distributed Architecture for Computing Context in Mobile Devices. M.Sc. Thesis.

[44] Orfanidis S.J. Introduction to Signal Processing.

[45] Gimon D., Gjoreski H., Kaluža B., Gams M. Using Accelerometers to Improve Position-Based Activity Recognition.

[46] Tolkiehn M., Atallah L., Lo B., Yang G.-Z. Direction Sensitive Fall Detection Using a Triaxial Accelerometer and a Barometric Pressure Sensor.

[47] Bersch S., Chislett C., Azzi D., Khusainov R., Briggs J. Activity Detection Using Frequency Analysis and Off-the-Shelf Devices: Fall Detection from Accelerometer Data.

[48] Teixeira T., Jung D., Dublon G., Savvides A. Recognizing Activities from Context and Arm Pose using Finite State Machines.

[49] Li Q., Stankovic J.A., Hanson M.A., Barth A.T. Accurate, Fast Fall Detection Using Gyroscopes and Accelerometer-Derived Posture Information.

[50] Dinh A., Teng D., Chen L., Shi Y. Implementation of a Physical Activity Monitoring System for The Elderly People With Built-in Vital Sign and Fall Detection.

[51] Zigel Y., Litvak D., Gannot I. (2009). A method for automatic fall detection of elderly people using floor vibrations and sound—Proof of concept on human mimicking doll falls. IEEE Trans. Biomed. Eng..

[52] Sekine M., Tamura T., Fujimoto T., Fukui Y. Classification of Walking Pattern Using Acceleration Waveform in Elderly People.

[53] Bao L., Intille S.S. Activity Recognition from User-Annotated Acceleration Data.

[54] Gjoreski H., Gams M., Chorbev I. 3-Axial Accelerometers Activity Recognition.

[55] (1980). Texas Instrument (TI). An Introduction to the Sampling Theorem.

[56] Antonsson E.K., Mann R.W. (1985). The frequency content of gait. J. Biomech..

[57] Jagos H., Oberzaucher J., Reichel M., Zagler W.L., Hlauschek W. (2010). A multimodal approach for insole motion measurement and analysis. Procedia Eng..

[58] Plasqui G., Bonomi A.G., Westerterp K.R. (2013). Daily physical activity assessment with accelerometers: New insights and validation studies. Obes. Rev..

[59] Bouten C.V., Koekkoek K.T., Verduin M., Kodde R. (1997). A triaxial accelerometer and portable data processing unit for the assessment of daily physical activity. IEEE Trans. Biomed. Eng..

[60] Celka P., Vetter R., Renevey P., Verjus C., Neuman V., Luprano J., Decotignie J.-D., Piguet C. (2005). Wearable biosensing: Signal processing and communication architectures issues. J. Telecommun. Inf. Technol..

[61] Wang W., Guo Y., Huang B., Zhao G. Analysis of Filtering Methods for 3D Acceleration Signals in Body Sensor Network.

[62] Tröster G. (2011b). Wearable Systems 1: Chapter 4 Time Series, Segmentation, DTW. www.ife.ee.ethz.ch/education/WS1_HS2012_04.pdf.

[63] Mannini A., Sabatini A.M. (2010). Machine learning methods for classifying human physical activity from on-body accelerometers. Sensors.

[64] Sixsmith A., Johnson N. (2004). A smart sensor to detect the falls of the elderly. IEEE Pervasive Comput..

[65] Figo D., Diniz P.C., Ferreira D.R., Cardoso J.M.P. (2010). Preprocessing techniques for context recognition from accelerometer data. Pers. Ubiquitous Comput..

[66] Pietka E. Expert Systems in Parameter Extraction of the ECG Signal.

[67] Keogh E., Smyth P. A Probabilistic Approach to Fast Pattern Matching in Time Series Databases.

[68] Keogh E., Chu S., Hart D., Pazzani M. An Online Algorithm for Segmenting Time Series.

[69] Himberg J., Korpiaho K., Mannila H., Tikanmaki J. Time Series Segmentation for Context Recognition in Mobile Devices.

[70] Ortiz J., Olaya A.G., Borrajo D. A Dynamic Sliding Window Approach for Activity Recognition.

[71] Casale P., Pujol O., Radeva P., Vitrià J., Sanches J.M., Hernández M. (2011). Human Activity Recognition from Accelerometer Data Using a Wearable Device.

[72] Achumba I., Bersch S., Khusainov R., Azzi D. On Time Series Sensor Data Segmentation for Fall and Activity Classification.

[73] Robinovitch S.N., Hayes W.C., McMahon T.A. (1997). Distribution of contact force during impact to the hip. Ann. Biomed. Eng..

[74] Irvine T. (2002). An Introduction to the Shock Response Spectrum.

[75] Li Y., Ho K.C., Popescu M. (2012). A microphone array system for automatic fall detection. IEEE Trans. Biomed. Eng..

[76] Bamberg S.J.M., Benbasat A.Y., Scarborough D.M. (2008). Gait analysis using a shoe-integrated wireless sensor system. IEEE Trans. Inf. Technol. Biomed..

[77] Im S., Kim I., Ahn S.C., Kim H. Automatic ADL Classification Using 3-axial Accelerometers and RFID Sensor.

[78] Crouter S.E., Clowers K.G., Bassett D.R. (2006). A novel method for using accelerometer data to predict energy expenditure. J. Appl. Physiol..

[79] Farringdon J., Moore A.J., Tilbury N., Church J., Biemond P.D. Wearable Sensor Badge and Sensor Jacket for Context Awareness.

[80] Jeong D.U., Kim S.J., Chung W.Y. Classification of Posture and Movement Using a 3-axis Accelerometer.

[81] Kawahara H.S.Y., Hisashi Kurasawa H.M., Aoyama T. A Context-Aware Collaborative Filtering Algorithm for Real World Oriented Content Delivery Service.

[82] Veltink P., Bussmann H., de Vries W., Martens W., van Lummel R. (1996). Detection of static and dynamic activities using uniaxial accelerometers. IEEE Trans. Rehabilit. Eng..

[83] Kern N., Schiele B., Schmidt A. (2007). Recognizing context for annotating a live life recording. Pers. Ubiquitous Comput..

[84] Jin G., Lee S., Lee T. (2007). Context awareness of human motion states using accelerometer. J. Med. Syst..

[85] Chambers G., Venkatesh S., West G., Bui H. Hierarchical Recognition of Intentional Human Gestures for Sports Video Annotation.

[86] Randell C., Muller H. (2000). Context Awareness by Analysing Accelerometer Data.

[87] Mathie M., Celler B., Lovell N., Coster A. (2004). Classification of basic daily movements using a triaxial accelerometer. Med. Biol. Eng. Comput..

[88] Nham B., Siangliulue K., Yeung S. (2008). Predicting Mode of Transport from iPhone Accelerometer Data.

[89] Chernbumroong S., Atkins A.S., Yu H. Activity Classification Using a Single Wrist-Worn Accelerometer.

[90] Dash M., Liu H. (1997). Feature selection for classification. Intell. Data Anal..

[91] Saeys Y., Inza I., Larranaga P. (2007). A review of feature selection techniques in bioinformatics. Bioinf. Rev..

[92] Arauzo-Azofra A., Aznarte J.L., Benítez J.M. (2011). Empirical study of feature selection methods based on individual feature evaluation for classification problems. Expert Syst. Appl..

[93] Sewell M. (2007). Feature Selection..

[94] Zongker D., Jain A. Algorithms for Feature Selection: An Evaluation.

[95] Fang H., Srinivasan R., Cook D.J. (2012). Feature selections for human activity recognition in smart home environments. Int. J. Innov. Comput. Inf. Control.

[96] Zigel Y., Cohen A. Text-Dependent Speaker Verification Using Feature Selection with Recognition Related Criterion.

[97] Pudil P., Novovicova J., Kittler J. (1994). Floating search methods in feature Selection. Pattern Recognition Lett..

[98] Maurer U., Smailagic A., Siewiorek D.P., Deisher M. Activity Recognition and Monitoring Using Multiple Sensors on Different Body Positions.

[99] Hall M.A. (1999). Correlation-based Feature Selection for Machine Learning. Ph.D. Thesis.

[100] Herrera E.P., Quiros R., Kaufmann H. (2007). Analysis of a kalman approach for a pedestrian positioning system in indoor environments. Lect. Notes Comput. Sci..

[101] Marschollek M., Rehwald A., Wolf K.-H., Gietzelt M., Nemitz G., Schwabedissen H.M., Schulze M. (2011). Sensors *vs.* experts—A performance comparison of sensor-based fall risk assessment *vs.* conventional assessment in a sample of geriatric patients. BMC J. Med. Informatics Decis. Making.

[102] Lee J.B., Mellifont R.B., Burkett B.J. (2010). The use of a single inertial sensor to identify stride, step, and stance durations of running gait. J. Sci. Med. Sport.

[103] Riva F., Toebes M.J.P., Pijnappel M., Stagni R., van Dieen J.H. (2013). Estimating fall risk with inertial sensors using gait stability measures that do not require step detection. Gait Posture.

[104] Diebel J. (2006). Representing Attitude: Euler Angles, Unit Quaternions, and Rotation Vectors..

[105] Ohtaki Y., Susumago M., Suzuki A., Sagawa K., Nagatomi R. (2005). Automatic classification of ambulatory movements and evaluation of energy consumptions utilizing accelerometers and a barometer. Microsyst. Technol..

[106] Godfrey A., Conway R., Meagher D., ÓLaighin G. (2008). Direct measurement of human movement by accelerometry. Med. Eng. Phys..

[107] Lord S.R., Clark R.D., Webster I.W. (1991). Postural stability and associated physiological factors in a population of aged persons. J. Gerontol..

[108] Gietzelt M., Nemitz G., Wolf K., Meyer H. (2009). A clinical study to assess fall risk using a single waist Accelerometer. J. Inform. Health Soc. Care.

[109] Oliver D., Britton M., Seed P., Martin F.C. (1997). Development and evaluation of evidence based risk assessment tool (STRATIFY) to predict which elderly inpatients will fall: Case-control and cohort studies. Brit. Med. J..

[110] Tinetti M.E., Williams T.F., Mayewski R. (1986). Fall risk index for elderly patients based on number of chronic disabilities. Amer. J. Med..

[111] Turcot K., Aissaoui R., Boivin K., Pelletier M., Hagemeister N., de Guise J.A. (2008). New accelerometric method to discriminate between asymptomatic subjects and patients with medial knee osteoarthritis during 3-D gait. IEEE. Trans. Biomed. Eng..

[112] Goldie P.A., Matyas T.A., Evans O.M. (2001). Gait after stroke: Initial deficit and changes in temporal patterns for each gait phase. Arch. Phys. Med. Rehabil..

[113] Liu T., Inoue Y., Shibata K.A. (2010). A wearable ground reaction force sensor system and its application to the measurement of extrinsic gait variability. Sensors.

[114] Chelius G., Braillon C., Pasquier M., Horvais N., Gibollet R.P., Espiau B., Coste C.A. (2011). A wearable sensor network for gait analysis: A six-day experiment of running through the desert. IEEE/ASME Trans. Mechatron..

[115] Zhu H.S., Maalej N., Webster J.G., Tompkins W.J., Bach-y-Rita P., Wertsch J.J. (1990). An umbilical data-acquisition system for measuring pressures between the foot and shoe. IEEE Trans. Biomed. Eng..

[116] Zhu H.S., Wertsch J.J., Harris G.F., Loftsgaarden J.D., Price M.B. (1991). Foot pressure distribution during walking and shuffling. Arch. Phys. Med. Rehabil..

[117] Hausdorff J.M., Ladin Z., Wei J.Y. (1995). Footswitch system for measurement of the temporal parameters of gait. J. Biomech..

[118] Hausdorff J.M., Zemany L., Peng C.-K., Goldberger A.L. (1999). Maturation of gait dynamics: Stride-to-stride variability and its temporal organization in children. J. Appl. Physiol..

[119] Pappas I.P., Popovic M., Keller T., Dietz V., Morari M. (2001). A reliable gait phase detection system. IEEE. Trans. Neural Syst. Reh. Eng..

[120] Pappas I.P., Keller T., Mangold S. A. Reliable Gyroscope Based Gait Phase Detection Sensor Embedded in a Shoe Insole.

[121] Chau T. (2001). A review of analytical techniques for gait data. Part 2: Neural network and wavelet methods. Gait Posture.

[122] Tucker C.A., White S.C. Neurocomputational Approaches to Pattern Recognition and Time-Series Analysis of Electromyographic Data Obtained during Treadmill Walking.

[123] Giansanti D. (2006). Investigation of fall-risk using a wearable device with accelerometers and rate gyroscopes. Physiol. Meas..

[124] Hausdorff J.M., Rios D.A., Edelberg H.K. (2001). Gait variability and fall risk in community-living older adults: A 1-year prospective study. Arch. Phys. Med. Rehabil..

[125] Zijlstra W., Aminian K. (2007). Mobility assessment in older people: New possibilities and challenges. Eur. J. Ageing.

[126] Mathie M.J. (2003). Monitoring and Interpreting Human Movement Patterns Using a Triaxial Accelerometer. Ph.D. Thesis.

[127] Scully P., Nurgiyatna N., Vaughan J., Wright P. Footprint imaging by guided path tomography.

[128] Tolstikov A., Biswas J., Chen-Khong T., Yap P. Eating Activity Primitives Detection—A Step Towards ADL Recognition.

[129] Naeem U., Bigham J., Wang J. Recognising Activities of Daily Life Using Hierarchical Plans.

[130] Makikawa M., Murakami D. Development of an Ambulatory Physical Activity and Behaviour Map Monitoring System.

[131] Ekelund U. Methods to Measure Physical Activity..

[132] Murphy S.L. (2009). Review of physical activity measurement using accelerometers in older adults: Considerations for research design and conduct. Prev. Med..

[133] Melanson E.L., Freedson P.S. (2006). Physical activity assessment: A review of methods. J. Crit. Rev. Food Sci. Nutr..

[134] Valanou E.M., Bamia C., Trichopoulou A. (2006). Methodology of physical-activity and energy-expenditure assessment: A review. J. Public Health.

[135] Troiano R.P. (2006). Translating accelerometer counts into energy expenditure: Advancing the quest. J. Appl. Physiol..

[136] Twomey N., Faul S.D., Marnane W.P.L. Comparison of Accelerometer-Based Energy Expenditure Estimation Algorithms.

[137] Machado-Rodrigues A.M., Coelho-E-Silva M.J., Mota J. (2011). Agreement in activity energy expenditure assessed by accelerometer and self-report in adolescents: Variation by sex, age, and weight status. J. Sports Sci..

[138] Ueno D.T., Sebastião É., Corazza D.I., Gobbi S. (2013). Methods for assessing physical activity: A systematic review focused on older adults. Braz. J. Kinanthropometry Hum. Perform..

[139] Morley R.E., Richter E.J., Klaesner J.W., Maluf K.S., Mueller M.J. (2001). In-shoe multisensory data acquisition system. IEEE Trans. Biomed. Eng..

[140] Keller P.A., Sinkovic S.P., Miles S.J. (1990). Skin dryness: A major factor in reducing incontinence dermatitis. Ostomy/Wound Manag..

[141] Mueller M.J., Strube M.J., Allen B.T. (1997). Therapeutic footwear can reduce plantar pressures in patients with diabetes and transmetatarsal amputation. Diabetes Care.

[142] Rozema A., Ulbrecht J.S., Pammer S.E., Cavanagh P.R. (1996). In-shoe plantar pressures during activities of daily living: Implications for therapeutic footwear design. Foot Ankle Int. J..

[143] Maluf K.S., Morley R.E., Richter E.J., Klaesner J.W., Mueller M.J. (2001). Monitoring in-shoe plantar pressures, temperature, and humidity: Reliability and validity of measures from a portable device. Arch. Phys. Med. Rehabil.

[144] Pataky Z., Faravel L., Da Silva J., Assal J.P. (2000). A new ambulatory foot pressure device for patients with sensory impairment: A system for continuous measurement of plantar pressure and a feed-back alarm. J. Biomech..

[145] Shu L., Hua T., Wang Y., Li Q., Feng D.D., Tao X. (2010). In-shoe plantar pressure measurement and analysis system based on fabric pressure sensing array. IEEE Trans. Inf. Technol. Biomed..

[146] Gupta K.O., Chatur P.N. (2012). ECG signal analysis and classification using data mining and artificial neural networks. Int. J. Emerg. Technol. Adv. Eng..

[147] Tayel M.B., El Bouridy M.E. ECG Images Classification using Artificial Neural Network Based on Several Feature Extraction Methods.

[148] Tadejko P., Rakowski W. Mathematical Morphology Based ECG Feature Extraction for the Purpose of Heartbeat Classification.

[149] Davis U.C. (1995). Monitoring Series No 1: Types of Monitoring. California Rangelands Research and Information Center (CRRIC). Agronomy and Range Science.

[150] Barakova E.I., Spink A.S., de Ruyter B., Noldus L.P.J.J. (2011). Trends in measuring human behavior and interaction. Pers. Ubiquit. Comput..

[151] Sobol-Shikler T., Robinson P. (2010). Classification of complex information: Inference of co-occurring affective states from their expressions in speech. IEEE Trans. Patt. Anal. Mach. Int..

[152] Dey K., Abowd G.D. Towards a Better Understanding of Context and Context-Awareness.

[153] Zhang M., Sawchuk A.A. Context-Aware Fall Detection Using a Bayesian Network.

[154] Rubenstein L.Z. (2006). Falls in older people: Epidemiology, risk factors and strategies for prevention. Age Ageing.

[155] Noury N. A Smart Sensor for the Remote Follow up of Activity and Fall Detection of the Elderly.

[156] Jantaraprim P., Phukpattaranont P., Limsakul C., Wongkittisuksa B. Improving the Accuracy of a Fall Detection Algorithm Using Free Fall Characteristics.

[157] Wu G., Xue S. (2008). Portable pre-impact fall detector with inertial sensors. IEEE Trans. Neural Syst. Rehabil. En..

[158] Allen F.R., Ambikairajah E., Lovell N.H., Celler B.G. An Adapted Gaussian Mixture Model Approach to Accelerometry-Based Movement Classification Using Time-Domain Features.

[159] Shuai Z., McClean S., Scotney B., Chaurasia P. Using Duration to Learn Activities of Daily Living in a Smart Home Environment.

[160] Nehmer J., Becker M., Karshmer A. Living Assistance Systems: An Ambient Intelligence Approach.

[161] Lord S.R., Sherrington C., Menz H.B. (2001). Falls in Older People: Risk Factors and Strategies for Prevention.

[162] Hausdorff J.M., Rios D.A., Edelberg H.K. (2001). Gait variability and fall risk in community-living older adults: A 1-year prospective study. Arch. Phys. Med. Rehabil..

[163] Kim E., Helal D.S. (2010). Human activity recognition and pattern discovery. IEEE Pervasive Comput. Mag..

[164] Lee P.H., Macfarlane D.J., Lam H.T. (2013). Factors associated with participant compliance in studies using accelerometers. Gait Posture.

[165] Tong L., Chen W., Song Q., Ge Y. A Research on Automatic Human Fall Detection Method Based on Wearable Inertial Force Information Acquisition System.

[166] Bourke A.K., O'brien J.V., Lyons G.M. (2007). Evaluation of a threshold-based tri-axial accelerometer fall detection algorithm. Gait Posture.

[167] Horak F.B. (2005). Postural orientation and equilibrium: What do we need to know about neural control of balance to prevent falls?. Age Ageing.

[168] Pei L., Guinnes R., Chen R., Liu J., Kuusniemi H., Chen Y., Chen L., Kaistinen J. (2013). Human behaviour cognition using smartphone sensors. Sensors.

[169] Ganyo M., Dunn M., Hope T. (2011). Ethical issues in the use of fall detectors. Ageing Soc..

